# CMTR1 promotes colorectal cancer cell growth and immune evasion by transcriptionally regulating STAT3

**DOI:** 10.1038/s41419-023-05767-3

**Published:** 2023-04-06

**Authors:** A-bin You, Hu Yang, Chun-ping Lai, Wen Lei, Lu Yang, Jia-lin Lin, Shun-cui Liu, Nan Ding, Feng Ye

**Affiliations:** 1 Department of Medical Oncology, Xiamen Key Laboratory of Antitumor Drug Transformation Research, the First Affiliated Hospital of Xiamen University, School of Medicine, Xiamen University, Xiamen, 361003 China; 2 The Third Clinical Medical College, Fujian Medical University, Fuzhou, 350122 China; 3 Department of Anesthesiology, the First Affiliated Hospital of Xiamen University, School of Medicine, Xiamen University, Xiamen, 361003 China

**Keywords:** Colon cancer, Immune evasion

## Abstract

CMTR1, also called IFN-stimulated gene 95 kDa protein (ISG95), is elevated by viral infection in a variety of cells. However, the functions of CMTR1 in colorectal cancer (CRC), especially its roles in tumorigenesis and immune regulation, remain unclear. Here, we first identified CMTR1 as a novel oncogene in colorectal cancer. Based on The Cancer Genome Atlas (TCGA) database exploration and human tissue microarray (TMA) analysis, we found that CMTR1 expression was markedly higher in CRC tissues than in adjacent normal tissues. High CMTR1 expression was correlated with poor prognosis in CRC patients. Knockdown (KD) of CMTR1 significantly suppressed cell proliferation and tumorigenicity both in vitro and in vivo, whereas overexpression of CMTR1 resulted in the opposite effects. KEGG pathway analysis revealed differential enrichment in the JAK/STAT signaling pathway in colorectal cancer cells with CMTR1 KD. Mechanistically, suppression of CMTR1 expression inhibited RNAPII recruitment to the transcription start site (TSS) of STAT3 and suppressed STAT3 expression and activation. Furthermore, the efficacy of PD1 blockade immunotherapy was prominently enhanced in the presence of CMTR1 KD via increased infiltration of CD8 + T cells into the tumor microenvironment. Overall, it appears that CMTR1 plays a key role in regulating tumor cell proliferation and antitumor immunity.

## Introduction

Colorectal cancer (CRC) is currently the third most prevalent cancer and a leading cause of all human cancer deaths worldwide [1]. Although marked progress has been made in treatments for CRC (including surgery, radiotherapy, chemotherapy, and targeted antiangiogenic therapy with VEGF or EGFR inhibitors), the prognosis of patients with CRC remains poor [2, 3]. Immune-checkpoint inhibitors (ICIs) have shown encouraging clinical responses in patients with CRC. Pembrolizumab was already approved by the US Food and Drug Administration (FDA) for the first-line treatment of patients with unresectable or metastatic microsatellite instability-high (MSI-H) CRC [4]. However, 95% of CRCs are microsatellite stable (MSS) or microsatellite instability-low (MSI-L), and the efficacy of ICIs in these cancers remains to be determined [5]. As a result, it is imperative to identify more effective molecular pathology-related diagnostic and prognostic indicators for CRC.

The 5′ ends of mRNAs in all eukaryotes and many viruses are modified with a 7-methylguanosine (m7G) cap (referred to as cap 0) [6]. In higher eukaryotes, the ribose 2′-O-hydroxyl sites in the first and second nucleotides at the 5′ ends of mRNAs, are methylated to generate the cap1 and cap2 structures, respectively [7]. A previous study indicated that in HeLa cells, almost every mRNA molecule has a cap1 structure, whereas only ~50% of mRNA molecules contain a cap2 structure [8]. Studies have shown that mRNA cap modifications have a series of functions. For example, cap0 recruits the translation initiation factor (eIF) 4 F complex (comprising eIF4E, 4 G, and 4 A) for cap-dependent translation, and cap 1 2′-O-methylation can further increase this translation-promoting effect [9]. In addition, cap 1 2′-O-methylation has been shown to protect the modified transcripts from exoribonuclease degradation or to serve as a marker of mRNA as self-RNA (host) versus nonself-RNA during viral infection [10].

Cap1 2′-O-methylation is catalyzed by cap methyltransferase 1 (CMTR1). CMTR1, also called IFN-stimulated gene 95 kDa protein (ISG95), is elevated by a viral infection and required to promote IFN-mediated induction of ISGs and the antiviral response [11]. Recent findings demonstrated that CMTR1 is recruited to transcription start sites (TSSs) and promotes ribosomal protein and histone gene expression, correlating with RNAPII binding and expression levels [12].

However, the functional roles of CMTR1 in tumorigenesis and immune regulation in CRC have not been explored. Here, we evaluated the expression and prognostic significance of CMTR1 in CRC through bioinformatics analysis of The Cancer Genome Atlas (TCGA) and human tissue microarray (TMA) data. Our results revealed that CMTR1 expression is higher in colorectal cancer tissues than in normal tissues, and that high CMTR1 expression is associated with poor prognosis in patients with CRC. Intriguingly, we found that CMTR1 controls cancer cell growth and antitumor immunity by binding to the TSS of STAT3. Moreover, inhibition of CMTR1 using siRNA reduced RNAPII binding to the STAT3 TSS and subsequently suppressed STAT3 expression and activation, providing important insights into the immune evasion of tumor cells in CRC.

## Materials and methods

### Cell culture and transfection

The human colorectal cancer cell lines HCT116, SW480, and RKO were kindly provided by Dajun Deng, who originally purchased these cells from ATCC [13], and cultured in RPMI 1640 medium. The human colorectal cancer cell line LoVo and the mouse colorectal cancer cell line MC38 were purchased from the National Infrastructure of Cell Line Resource (Beijing, China), and maintained in DMEM medium. All cells were maintained in a culture medium supplemented with 10% fetal bovine serum (FBS; BI, Israel) and 100 U/mL penicillin/streptomycin (BI, Israel) at 37 °C in a humidified incubator with 5% CO_2_. In addition, mycoplasma contamination was ruled out using a PCR-based method.

For CMTR1 and STAT3 knockdown, siRNA targeting human CMTR1 and STAT3 mRNA sequences obtained from RiboBio Co., Ltd. (Guangzhou, China) were used. CRC cells were seeded in 6 cm plates before transfection. When the cells reached a confluence of 60–80%, they were transfected with siRNA (50 nM) with Lipofectamine 2000 (Thermo Fisher Scientific) according to the manufacturer’s instructions. Eight hours after transfection, the supernatant was replaced, and cells were cultured for another 48 h.

The plasmids p3xflag-cmv-hCMTR1 and vector control were synthesized by Syngenbio Co., LTD. (Beijing, China). The LV3(H1/GFP&Puro)-sh-CMTR1, the LV3(H1/GFP&Puro)-sh-Cmtr1, and empty vector plasmids were constructed by GenePharma Co. (Shanghai, China). To construct lentivirus-stable transfection cell lines, HEK293T cells were seeded in 6 cm plates. When the cell confluence reached 40%, they were transfected with the LV3(H1/GFP&Puro)-sh-CMTR1, the LV3(H1/GFP&Puro)-sh-Cmtr1 and empty vector plasmids using lentivirus packaging vectors pMDL, VSVG, and REV at a ratio of 10:5:3:2. After 48 h transfection, the cell supernatant was collected and filtered by a 0.45 μm needle filter, and then used to infect RKO or MC38 cells. Afterward, the infected cells were cultured in a medium containing 1 μg/mL puromycin (Sigma, St. Louis, USA) for at least 2 weeks to obtain stably transfected cells. All sequence information is presented in Table S1.

### RNA extraction and real-time PCR analysis of gene expression

Total RNA was isolated using an Ultrapure RNA Kit (CWBIO, Jiangsu, China) according to the manufacturer’s instructions. The quality and concentration of the RNA samples were determined with a NanoDrop 2000 system (Thermo Fisher Scientific, Inc.). Qualified RNA samples were reverse transcribed into complementary DNA using GoScript™ Reverse Transcription Mix (Promega, random primers). Quantitative RT‒PCR (RT‒qPCR) was performed using a StepOne Real-Time PCR System (Applied Biosystems, Foster City, CA, USA) and SYBR Green PCR master mix reagents (FastStart Universal SYBR Green Master, Roche, Mannheim, Germany). Expression levels were calculated using the 2^−ΔΔCt^ method. Primer sequence information is presented in Table S1.

### RNA sequencing (RNA-seq)

For genome-wide transcriptome profiling, two libraries were generated from samples of total RNA extracted from scramble RNA control, and CMTR1 KD RKO cells using the NEBNext® Ultra™ Directional RNA Library Prep Kit for Illumina(E7420L). Paired-end sequencing was performed on the Illumina HiSeq 3000 platform at Amogene Biotech Co., Ltd. TopHat (http://ccb.jhu.edu/software/tophat/index.shtml) was used to map the cleaned reads to the human hg19 reference genome [14]. mRNA data obtained by transcriptome sequencing were analysed statistically with the *t*-test and differences were corrected with an RVM model. Significantly differentially expressed mRNAs between CMTR1 KD and scramble control cells were identified. The CMTR1-regulated gene program was identified by evaluating the fold changes (FCs) in gene FPKM (fragments per kilobase per million mapped reads) values between si-Ctrl and si-CMTR1-transfected cells (with FC ≥1.2 indicating a significant difference). Raw data were deposited in the Gene Expression Omnibus under accession number GSE220040.

### Cell proliferation assays

For the Cell Counting Kit-8 (CCK8) assay, treated cells were seeded into 96-well plates at 2000 cells/well for various lengths of time. RPMI 1640 medium (100 μl) and CCK8 assay solution (Dojindo, Japan) (10 μl) were added to each well, and the plates were incubated for 2 h. Then, the absorbance at 450 nm was measured every 24 h with a microplate reader at 450 nm. For the colony formation assay, equal numbers of treated cells were seeded into 6-well plates and cultured at 37 °C for 2–3 weeks until colonies developed. The cells were fixed with 4% paraformaldehyde for 15 min and stained with 0.1% crystal violet for 15 min. Then, macroscopic colonies were photographed and counted. For the EdU (Beyotime, China) incorporation assay, treated cells were plated in 24-well plates at 8000 cells/well, 10 μM EdU was added, and the plates were incubated for 2 h. Subsequently, the cells were fixed and permeabilized with 4% paraformaldehyde and 0.3% Triton X-100, respectively. After incubation with click reaction buffer in the dark for 30 min, the cells were stained with Hoechst 33342. Images were acquired using a fluorescence microscope.

### Western blotting analysis

Western blotting analysis was performed as previously described [13]. In brief, cells were lysed with RIPA buffer containing protease and phosphatase inhibitors. Protein concentration were measured with BCA reagent (Beyotime, China). At least 20 μg of the sample was used to detect protein expression. The antibodies used in the experiments were as follows: anti-CMTR1 (#ab70386; Abcam, UK); anti-CDKN1A, anti-CDK6, anti-CCND1, anti-STAT3, anti-p-STAT3 (Y705) (#A19094, #A0106, #A19038, #A19566, #AP0705; ABclonal, China); and anti-GAPDH (#60004-1; Protein Tech, China).

### Chromatin immunoprecipitation (ChIP) assay

For the ChIP assay, cells were fixed at room temperature (RT) for 10 min with 1% formaldehyde. After fixation, cells were collected and lysed in cell lysis buffer (1% SDS, 10 mM EDTA, 50 mM Tris-HCl) containing 1X protease inhibitor cocktail. Chromatin was sheared into 100–500 bp fragments by sonicating the lysates on ice using a Bioruptor, followed by centrifugation at maximum speed for 15 minutes. Five microlitres of supernatant was retained as input DNA for subsequent PCR analysis. The remaining supernatant was incubated with a CMTR1 (#ab70386; Abcam, UK) or RNAPII (#ab5131; Abcam, UK) specific antibody or with nonspecific IgG overnight at 4 °C. Subsequently, protein G magnetic beads (Bio-Rad, 161-4023) were added and incubated for another 4 h at 4 °C. Following immunoprecipitation, the complexes were washed once with TSE1 buffer (0.1% SDS, 1%Triton X-100, 2 mM EDTA, 150 mM NaCl, 20 mM Tris-HCl), once with TSE2 buffer (0.1% SDS, 1%Triton X-100, 2 mM EDTA, 400 mM NaCl, 20 mM Tris-HCl), and twice with TE buffer (20 mM Tris-HCl, 1 mM EDTA). The immunoprecipitants were then de-crosslinked at 65 °C overnight. Finally, the immunoprecipitated DNA was purified with QIAquick Spin Columns (Qiagen) and tested by qPCR.

### Animals and in vivo experiments

Both male BALB/c nude mice and male C57BL/6 J mice aged 4–6 weeks were purchased from the Animal Research Center, Beijing, China. To investigate the effect of CMTR1 on cancer cell growth in vivo, two groups (6 mice/group) of randomly selected BALB/C nude mice were subcutaneously implanted with 1 × 10^7^ sh-Ctrl, sh-CMTR1-infected RKO cells suspended in PBS. Tumor growth was monitored daily. After 3 weeks, the mice were sacrificed, and the tumors were harvested and weighed. For anti-PD1 antibody treatment, MC38 cells (2 × 10^6^) with or without CMTR1 KD were injected subcutaneously into the flank area of randomly selected C57BL/6 J mice. Anti-PD1 antibodies (BioXcell, Cat#BE0146, 200 µg per mouse) or InVivoPlus rat IgG2a isotype control (BioXcell, Cat#BP0089, 200 µg per mouse) were injected intraperitoneally every four days for a total of four injections. The mice were sacrificed, and the CRC tumor tissues were fixed with 10% formaldehyde for subsequent immunohistochemical staining. Ethical approval was provided by the First Affiliated Hospital of Xiamen University (Xiamen, China) research ethics committee. We used the ARRIVE1 checklist when writing our report [15].

### Cohort and immunohistochemistry

A tissue microarray containing 73 paired human colon cancer tissues (Cat No. COC1601) was purchased from Shanghai Superbiotek Pharmaceutical Technology Co., Ltd. (Shanghai). All studies involving human samples were approved by the ethics committee of the First Affiliated Hospital of Xiamen University. Informed consent was obtained from each patient prior to inclusion in the study. Immunohistochemistry was performed as previously described with a slight modification [16]. Briefly, tumor tissues were fixed with 4% formaldehyde and cut into 4-5 mm slices. The tissue slides were subjected to EDTA antigen retrieval and blocked with 3% H2O2 for 10 min at room temperature. The tissues in the COC1601 array were immunostained with an anti-CMTR1 antibody (#IHC-00411-T, Bethyl, USA), and mouse xenograft tumor tissues were immunostained with an anti-CD8 antibody (#GB13429, Servicebio, China) at 4 °C overnight. Subsequently, the slice was incubated with a secondary antibody for 1 h at room temperature, and staining was visualized with DAB.

### Statistical analysis

All data are presented as the mean ± SD of at least three independent experiments. Data were analysed using SPSS 22. 0 software (SPSS, Inc., Chicago IL, USA) and GraphPad Prism 8 (GraphPad Software, Inc., La Jolla, CA, USA). Statistical analysis methods included Student’s *t*-test, one-way analysis of variance (ANOVA), and two-way ANOVA. A *p* value of <0.05 was considered statistically significant.

## Results

### CMTR1 expression positively correlates with the progression of colorectal cancer

**Fig. 1:**
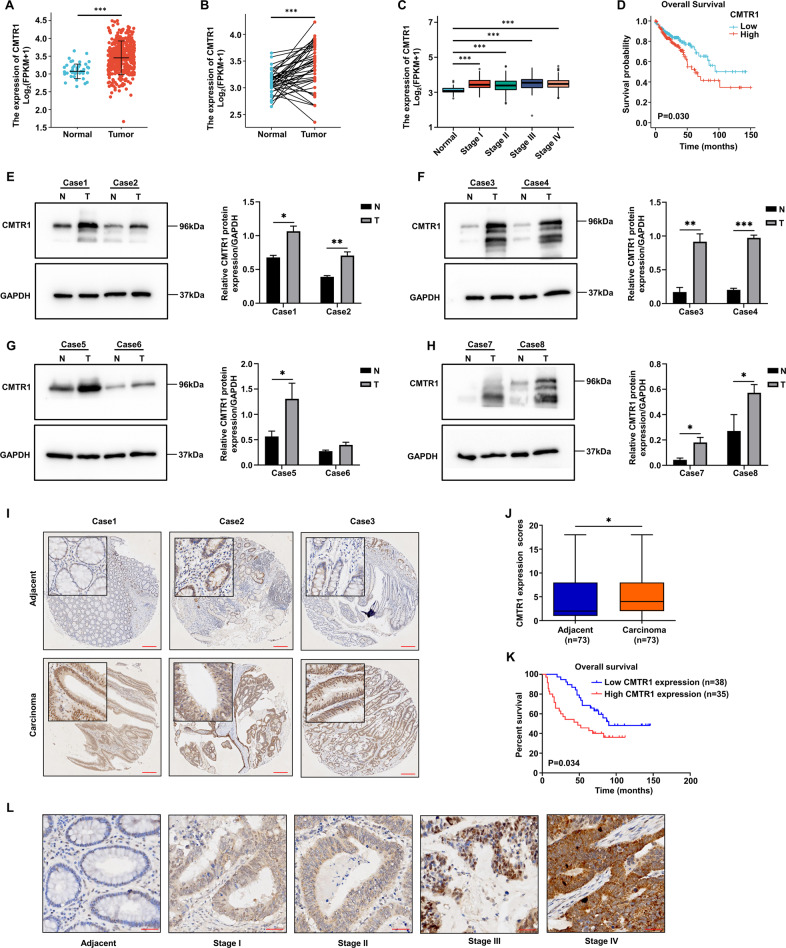
CMTR1 expression positively correlates with the progression of colorectal cancer. **A** Analysis of CMTR1 expression in human colon cancer tissues (*n* = 480) and adjacent normal tissues (*n* = 41) based on a TCGA dataset. **B** Analysis of CMTR1 expression in paired human colon cancer tissues (*n* = 41) and adjacent normal tissues (*n* = 41) based on a TCGA dataset. **C** Relative expression of CMTR1 in normal individuals and in COAD patients with tumors of different pathologic stages. **D** Kaplan‒Meier survival curves of overall survival (OS) in the TCGA-COAD cohort. **E**–**H** The CMTR1 protein levels in CRC tumor tissues (T) and matched adjacent normal tissues (N) were measured by western blotting. **I** Representative images of immunohistochemical staining for CMTR1 in CRC tumor tissues (*n* = 71) and matched adjacent normal tissues (scale bar, 200 μm). **J** CMTR1 expression scores are shown on box plots. The data were analysed using the Kruskal‒Wallis test. **K** Kaplan‒Meier survival curves of overall survival (OS) in our patient cohort stratified by CMTR1 staining. **L** Representative images of immunohistochemical staining to evaluate CMTR1 protein expression in colorectal cancer tissues at four clinical stages (scale bar, 40 μm). *P < 0.05, **P < 0.01, and ***P < 0.001.

We first compared the mRNA expression of CMTR1 in tumor and adjacent normal tissues from the TCGA-COAD cohort to explore the specific role of CMTR1. The results indicated that the mRNA expression of CMTR1 was significantly higher in tumor tissues than in adjacent normal tissues (Fig. 1A), and paired analysis further revealed that the mRNA expression of CMTR1 in tumor tissues was elevated (Fig. 1B). According to the gene expression profile and clinical data obtained from the TCGA-COAD dataset, the expression of CMTR1 was significantly higher in tumor tissues than in normal controls and increased with pathologic stage (Fig. 1C), and Kaplan‒Meier survival analysis indicated that high CMTR1 expression was correlated with poor prognosis (Fig. 1D). We further examined CMTR1 protein expression in CRC patient tissues by western blotting. Consistent with the TCGA analysis results, CRC tumor tissues had higher CMTR1 protein expression than adjacent normal tissues (Fig. 1E–H).

To further investigate the relationship between the CMTR1 expression level and clinicopathological characteristics in CRC, a tissue microarray containing 73 pairs of patient-derived CRC and adjacent normal tissues was used to assess the expression of CMTR1. Immunohistochemical (IHC) staining demonstrated that CRC tumor tissues exhibited significantly higher CMTR1 protein expression than adjacent normal tissues, and that CMTR1 was localized mainly in the nucleus (Fig. 1I, J). Next, we analysed the prognostic role of CMTR1 expression in CRC. The overall survival of patients with relatively high levels of CMTR1 was less favorable than that of patients with low levels of CMTR1 (Fig. 1K). In addition, the correlation between CMTR1 protein expression and tumor pathologic stage was verified. CMTR1 protein expression was generally lower in adjacent normal tissues and higher in tumor tissues, and increased with the pathologic stage (Fig. 1L).

### CMTR1 regulates the expression of a group of genes involved in the cell cycle

**Fig. 2:**
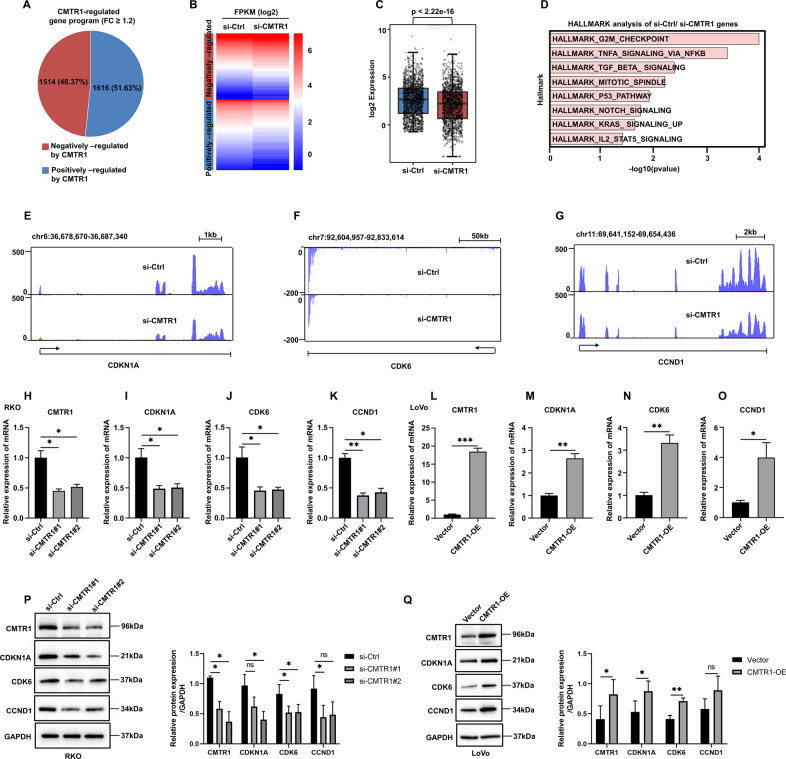
CMTR1 regulates the expression of a group of genes involved in the cell cycle. **A** RNA-seq was conducted on RKO cells transfected with control siRNA (si-Ctrl) or CMTR1 siRNA (si-CMTR1). A pie chart was used to show genes positively and negatively regulated by CMTR1. **B**, **C** The expression levels of genes positively and negatively regulated by CMTR1 are displayed in a heatmap and a box plot. **D** Hallmark gene set analysis revealed the gene term with the greatest enrichment of genes positively regulated by CMTR1. **E**–**G** Graphical presentation of the effects of CMTR1 on representative cell cycle-related genes, such as CDKN1A, CDK6, and CCND1, as determined by RNA-seq. **H**–**Q** Expression of CDKN1A, CDK6, and CCND1, measured by RT‒qPCR and western blotting in CMTR1 knockdown and CMTR1-overexpressing cells. *P < 0.05, **P < 0.01, and ***P < 0.001.

To investigate the potential functions of CMTR1 in CRC, we performed a whole transcriptome analysis of RKO cells after CMTR1 knockdown using RNA sequencing. The results showed that 1616 genes were positively regulated and 1514 genes were negatively regulated by CMTR1 (fold change (FC) ≥1.2) (Fig. 2A). The expression of these CMTR1-regulated genes visualized in a heatmap and a box plot (Fig. 2B, C). Hallmark gene set analysis revealed that genes positively regulated by CMTR1 were enriched mainly in the “G2M CHECKPOINT” term (Fig. 2D). The effects of CMTR1 on representative cell cycle-related genes identified by RNA sequencing, such as CDKN1A, CDK6, and CCND1, are shown in Figure 2E–G. The results demonstrated that the knockdown of CMTR1 downregulated the expression of cell cycle-related genes, and this effect was further confirmed by RT‒qPCR and western blotting analysis in CMTR1 knockdown and CMTR1-overexpressing cells (Fig. 2H–Q and Figs. S1A–E, S2A–E). Furthermore, the flow-cytometry analysis showed that CMTR1 could regulate cell cycle progression (Fig. S3A, B). Therefore, CMTR1 appears to be necessary for cell cycle progression.

### CMTR1 promotes colorectal cancer cell growth both in vitro and in vivo

**Fig. 3:**
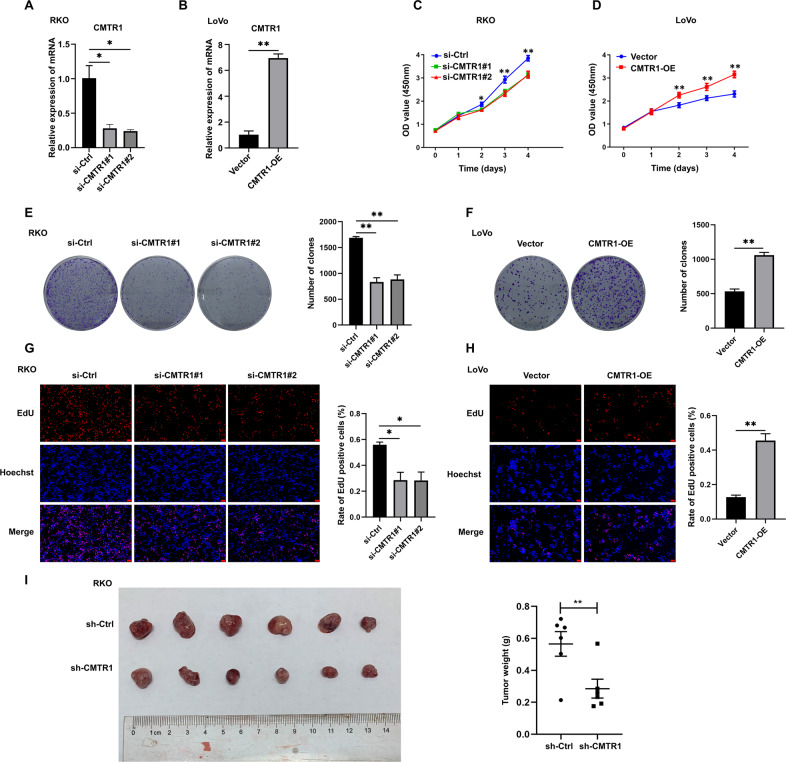
CMTR1 promotes colorectal cancer cell growth both in vitro and in vivo. **A**, **B** CMTR1 mRNA expression was measured by RT‒qPCR in CMTR1 knockdown and CMTR1-overexpressing cells. **C**–**H** CCK8, colony formation, and EdU incorporation assays were performed to evaluate cell proliferation (scale bar, 50 μm). **I** Subcutaneous tumorigenesis in mice was evaluated after CMTR1 knockdown. *P < 0.05, **P < 0.01, and ***P < 0.001.

To characterize the roles of CMTR1 in cell proliferation, we knocked down and overexpressed CMTR1 in several colorectal cancer cell lines (Fig. 3A, B and Figs. S1A, S2A). The CCK8 assay results revealed that CMTR1 knockdown effectively decreased the proliferation and viability of RKO and HCT116 cells, while upregulation of CMTR1 expression significantly enhanced the proliferation ability of LoVo and SW480 cells (Fig. 3C, D and Figs. S1G, S2G). The colony formation assay further demonstrated that the number of colonies formed by colorectal cancer cells was obviously reduced by knockdown and increased by overexpression of CMTR1 (Fig. 3E, F and Figs. S1H, S2H). In addition, as a measure of cell proliferation, the EdU incorporation assay indicated that the knockdown of CMTR1 was greatly reduced, but overexpression of CMTR1 increased the percentage of EdU-positive cells (Fig. 3G, H). To evaluate the effect of CMTR1 on tumor growth in vivo, we subcutaneously implanted RKO cells infected with lentivirus expressing control shRNA or shRNA targeting CMTR1 into the left inguinal region of nude mice. We found that CMTR1 knockdown significantly decreased tumorigenesis compared to that resulting from injection of control shRNA-transduced cells (Fig. 3I). Taken together, our data revealed a functional role of CMTR1 in promoting colorectal cancer cell growth both in vitro and in vivo.

### CMTR1 affects the expression of inflammatory factors in colorectal cancer cells by transcriptionally regulating STAT3

**Fig. 4:**
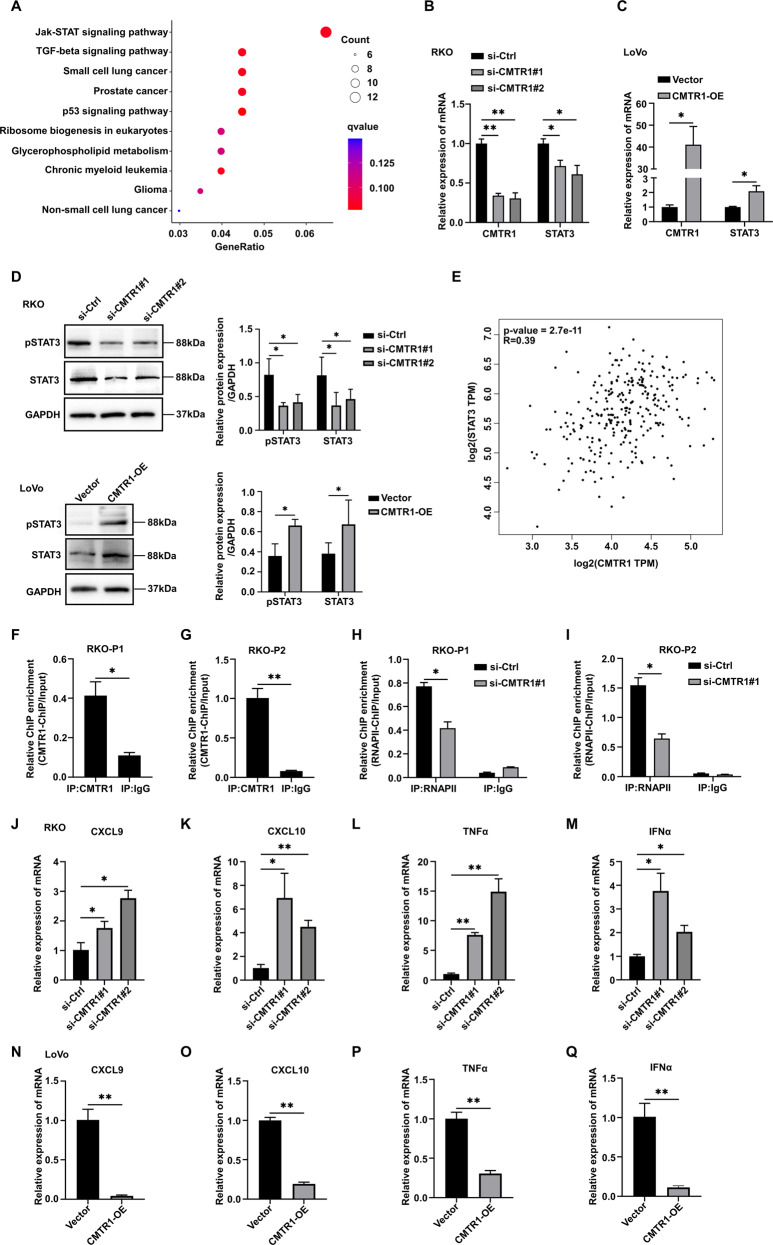
CMTR1 affects the expression of inflammatory factors in colorectal cancer cells by transcriptionally regulating STAT3. **A** KEGG pathway analysis revealed that genes differentially regulated by CMTR1 knockdown were significantly enriched in the JAK-STAT signaling pathway. **B**, **C** STAT3 mRNA expression was measured by RT‒qPCR in CMTR1 knockdown and CMTR1-overexpressing cells. **D** STAT3 and pSTAT3 protein expression levels were assessed by western blotting after knockdown and overexpression of CMTR1. **E** Correlation analysis of CMTR1 and STAT3 expression using public TCGA transcriptome datasets via the gene expression profiling interactive analysis (GEPIA) server. **F**, **G** RKO cells were subjected to ChIP analysis with control IgG or an anti-CMTR1 antibody, followed by RT‒qPCR analysis using two primer sets (P1 and P2) targeting the two predicted CMTR1 binding sites in the STAT3 promoter region. **H**, **I** After knockdown of CMTR1, RKO cells were subjected to ChIP analysis with control IgG or an anti-RNAPII antibody, followed by RT‒qPC analysis using two primer sets (P1 and P2) targeting the two predicted RNAPII binding sites in the STAT3 promoter region. **J**–**Q** Relative mRNA levels of inflammatory mediators after knockdown and overexpression of CMTR1. *P < 0.05, **P < 0.01, and ***P < 0.001.

Several oncogenic signaling pathways, including the JAK/STAT [17], MAPK/ERK [18], WNT [19], PI3K/AKT [20], and Notch pathways [21], are involved in the regulation of cancer cell proliferation. In this study, KEGG pathway analysis showed that the expression of JAK/STAT signaling pathway-related genes, but not MAPK/ERK, WNT, PI3K/AKT, or Notch signaling pathway-related genes, was affected in RKO cells with CMTR1 knockdown (Fig. 4A). STAT3 is an important transcription factor in the JAK/STAT3 pathway, and an increase in STAT3 phosphorylation can increase its activity and promote the proliferation of colorectal cancer cells [17]. The results of RT‒qPCR analysis indicated that the STAT3 mRNA level was reduced after CMTR1 knockdown (Fig. 4B), and increased after CMTR1 overexpression (Fig. 4C). Moreover, western blotting showed that knockdown of CMTR1 decreased and overexpression of CMTR1 increased the levels of both phospho-STAT3 (Tyr705) (pSTAT3) and total STAT3 (Fig. 4D). By using the Gene Expression Profiling Interactive Analysis (GEPIA) server [22], we further observed a positive correlation between the mRNA levels of CMTR1 and STAT3 in COAD (*R* = 0.39, *p* < 0.001) (Fig. 4E).

As published previously, CMTR1 was found to be located mainly at the 5’ end of genes close to the TSS (transcription start site), and over 700 genes were occupied by CMTR1 within 500 bp of the TSS (designated “CMTR1-enriched genes”) [12]. By reviewing the ChIP-seq results presented in the text, we found that STAT3 was also among the CMTR1-enriched genes. Subsequently, ChIP‒qPCR was performed with an anti-CMTR1 antibody to confirm that CMTR1 bound strongly to the TSS of STAT3 (Fig. 4F, G). The study referenced above also demonstrated that CMTR1 was colocalized with RNAPII in coding and noncoding genes adjacent to TSSs and that CMTR1 could influence RNAPII recruitment or retention during the early stages of transcription [12]. Therefore, we explored whether CMTR1 knockdown can affect the recruitment of RNAPII to the STAT3 gene. The ChIP‒qPCR results revealed that the binding of RNAPII to the STAT3 TSS was reduced after CMTR1 knockdown, indicating a potential role for CMTR1 in directly regulating STAT3 transcription (Fig. 4H, I).

It has been reported that IFNα-activated STAT3 inhibits STAT1-dependent gene activation, thereby downregulating IFNα-mediated induction of inflammatory mediators such as the chemokines CXCL9 and CXCL10 [23]. Another study showed that pSTAT3 interacted with STAT1 to inhibit the type I IFN response [24]. In this study, we explored whether the expression of inflammatory mediators is regulated by CMTR1. The RT‒qPCR results indicated that knockdown of CMTR1 expression by siRNA increased the expression of chemokines and proinflammatory factors, including CXCL9, CXCL10, TNFα, and IFNα (Fig. 4J–M and Fig. S1I–L). However, overexpression of CMTR1 had the opposite effect (Fig. 4N–Q and Fig. S2I–L).

### CMTR1-regulated tumor cell proliferation and immune responses are STAT3-dependent

**Fig. 5:**
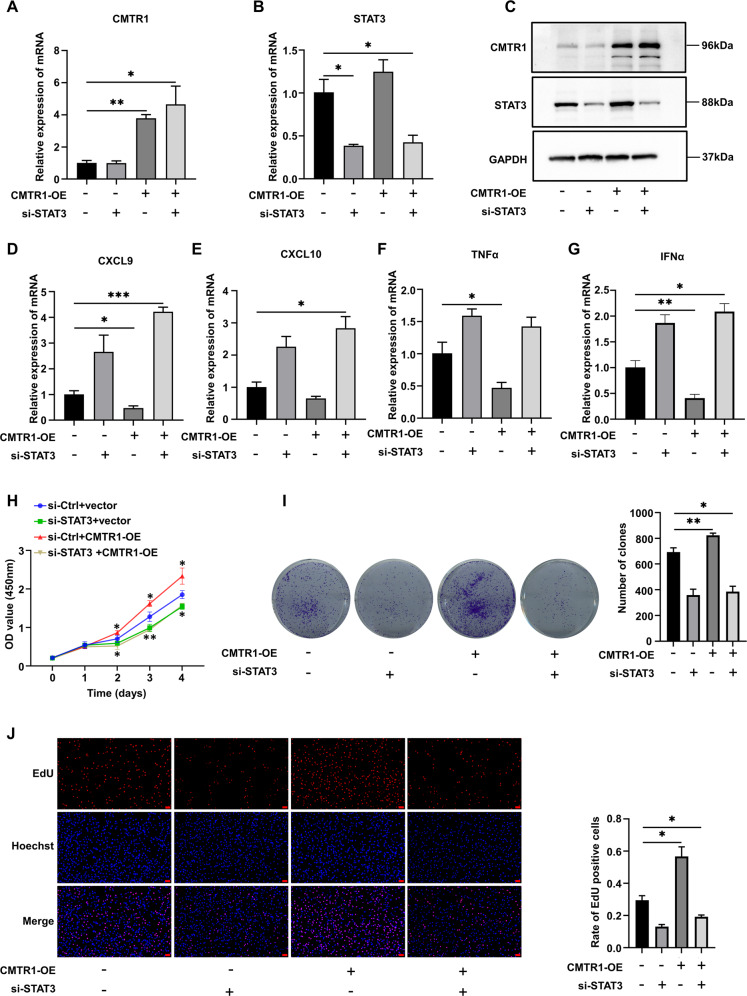
CMTR1-regulated tumor cell proliferation and immune responses are STAT3-dependent. **A**–**G** Changes of STAT3 knockdown on CMTR1-induced inflammatory mediators. **H**–**J** Effect of STAT3 knockdown on CMTR1-induced tumor cell proliferation, as assessed by CCK8, colony formation, and EdU incorporation assays (scale bar, 50 μm). *P < 0.05, **P < 0.01, and ***P < 0.001.

To address whether CMTR1 regulates tumor cell proliferation and immune response through STAT3, we overexpressed CMTR1 in STAT3-knockdown RKO cells (Fig. 5A–C). The RKO cells were first transfected with STAT3 siRNA for 24 h, and then transfected with p3xflag-cmv-hCMTR1 for another 48 h. The results showed that knockdown of STAT3 expression by siRNA decreased RKO cell proliferation (Fig. 5H–J), but increased the expression of chemokines (Fig. 5D, E) and proinflammatory factors (Fig. 5F, G) and completely reversed the CMTR1-mediated induction of tumor cell proliferation and immune responses. These results implied that tumor cell proliferation and immune responses regulated by CMTR1 were STAT3-dependent.

### CMTR1 knockdown enhances the efficacy of PD1 blockade immunotherapy in colorectal cancer

**Fig. 6:**
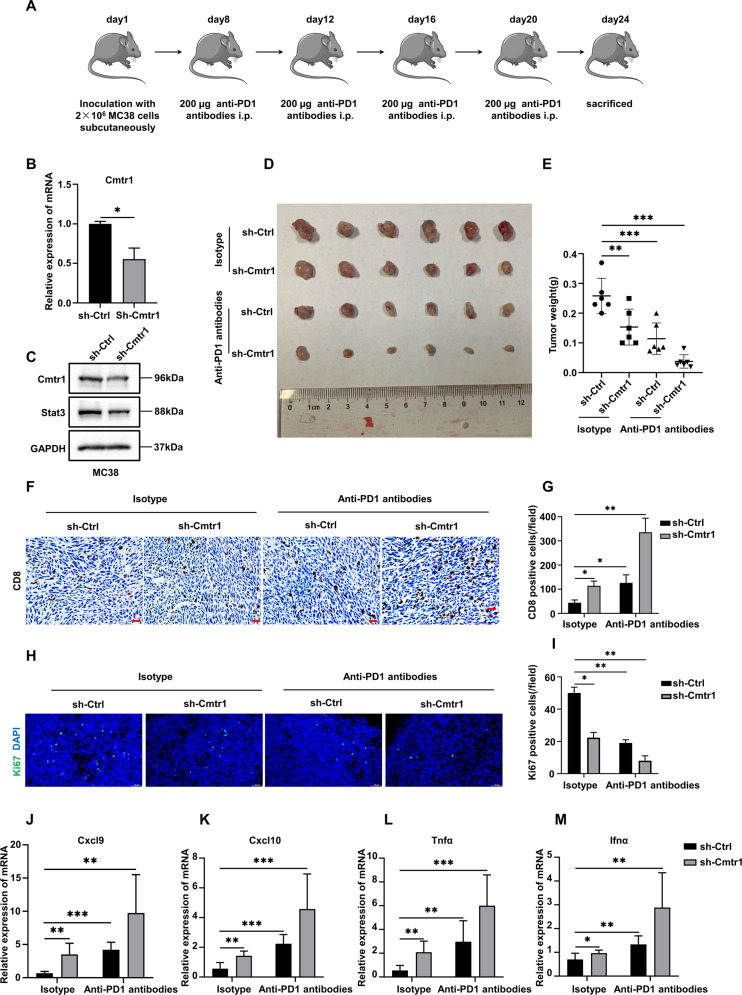
CMTR1 knockdown enhances the efficacy of PD1 blockade immunotherapy in colorectal cancer. **A** Schematic diagram showing the time point of anti-PD1 antibody treatment and analysis of tissues from C57BL/6 J mice implanted with MC38 cells. **B**, **C** After the knockdown of CMTR1 expression using shRNA, the expression of CMTR1 was measured by RT‒qPCR and western blotting. **D**, **E** Representative pictures of MC38 cell-derived tumors in C57BL/6 J mice, and tumor weights indicating the effects of CMTR1 knockdown or in combination with PD1. **F**, **G** Immunohistochemical staining and semi-quantitative analysis of CD8 positive T cells in tumors from mice indicating the effects of CMTR1 knockdown alone or in combination with PD1 blockade therapy. Scale bar: 50 µm. **H**, **I** Immunofluorescent staining and quantification of ki67 in tumors from mice upon CMTR1 knockdown or in combination with PD1. Scale bar: 50 µm. **J**–**M** Relative mRNA levels of inflammatory mediators in tumors from mice treated as indicated. *P < 0.05, **P < 0.01, and ***P < 0.001.

Since CMTR1 knockdown reduced STAT3 expression and activation, we further examined whether CMTR1 knockdown can enhance the efficacy of PD1 blockade immunotherapy in colorectal cancer by activating tumor cell-intrinsic type 1 IFN signaling. We first generated a murine MC38 colorectal cancer cell line with stable CMTR1 knockdown (sh-CMTR1), and sh-Ctrl/sh-CMTR1 cells were then transplanted subcutaneously into mice (Fig. 6A–C). The results showed that anti-PD1 treatment moderately reduced the growth of tumors derived from sh-Ctrl cells, while CMTR1 knockdown further significantly enhanced the inhibition of tumor growth mediated by anti-PD1 treatment (Fig. 6D, E). Immunostaining revealed that the combination of CMTR1 knockdown and anti-PD1 treatment resulted in significantly increased recruitment of CD8 + T cells into the tumor microenvironment compared with that resulting from anti-PD1 treatment or CMTR1 knockdown alone (Fig. 6F, G). Consistently, the combination of CMTR1 knockdown and anti-PD1 treatment significantly inhibited CRC proliferation compared with CMTR1 knockdown or anti-PD1 treatment alone, as determined by immunostaining of ki67 (Fig. 6H, I). Subsequently, we examined the expression of inflammatory factors in tumor tissues of mice in different treatment groups. The results demonstrated that anti-PD1 treatment promoted the expression of chemokines and proinflammatory factors, and that CMTR1 knockdown further increased these effects (Fig. 6J–M).

## Discussion

Increasing evidence indicates that tumor cell proliferation and immune evasion play an important role in tumorigenesis and tumor development [25, 26]. CMTR1 was first identified as an interferon-regulated gene, and plays a pivotal role in gene expression and innate immunity [27, 28]. In this study, we found that CMTR1 expression was higher in colorectal cancer tissues than in normal tissues and that CMTR1 expression was associated with poor prognosis. Knockdown of CMTR1 inhibited cancer cell growth both in vitro and in vivo, with a concomitant reduction in the expression of cell cycle-related genes, such as CDKN1A, CDK6, and CCND1. Therefore, CMTR1 appears to play a role in promoting tumorigenesis.

Interferons (IFNs) are cytokines that activate the heterotrimeric transcription factor complex ISGF3 (IFN-stimulated gene factor 3) via the JAK-STAT pathway. Activated ISGF3 translocates to the nucleus and binds to IFN response elements in the promoters of ISGs, which encode proteins that exhibit their antitumor activity [29–32]. During the type I IFN response, the mRNA transcripts of some ISGs require CMTR1 to evade sensing and translational inhibition by interferon-induced proteins with tetratricopeptide repeats 1(IFIT1) [33]. An early study indicated that cap1-deficient cellular transcripts in CMTR1 KD cells were recognized by RIG-I to activate IFN signaling [34].

Transcriptomic analysis revealed differential enrichment in the JAK/STAT signaling pathway in colorectal cancer cells with CMTR1 KD. STAT3 is a key downstream regulator of the JAK-STAT pathway that is involved in the inflammatory immune response [35]. Aberrant activation of STAT3 has been reported in many human solid tumors, including colorectal cancer [36]. Moreover, STAT3 creates an immunosuppressive environment by regulating the expression of immune factors and recruiting immunosuppressive cells [37]. Here, our study revealed a novel molecular mechanism by which CMTR1 regulates STAT3 expression and activation at the transcriptional level. We revealed that CMTR1 synergistically controlled tumor cell proliferation and antitumor immunity by binding to the TSS of STAT3. More importantly, the knockdown of CMTR1 reduced RNAPII recruitment to the STAT3 TSS and suppressed STAT3 expression and activation. In addition, we found that knockdown of CMTR1 increased the expression of chemokines and proinflammatory factors, including CXCL9, CXCL10, TNFα, and IFNα, while overexpression of CMTR1 resulted in the opposite effect.

Due to the lack of CD8 + T-cell infiltration, many tumors are resistant to PD1 blockade immunotherapy [38]. PD1 blockade was reported to be effective in only 1 of 33 patients with colorectal cancer, and this patient was hypothesized to have a mismatch repair-deficient (dMMR) or microsatellite instability-high (MSI-H) CRC [39]. dMMR/MSI-H CRC usually exhibits prominent lymphocytic infiltration in and around the tumor [40]. However, only 5% of CRC cases are dMMR/MSI-H CRC. In this study, we found that CMTR1 knockdown significantly enhanced the tumor cell response to PD1 blockade by increasing the recruitment of CD8 + T cells. Our work revealed that CMTR1 might be an important regulator of the immunosuppressive microenvironment of colorectal cancer. Although CMTR1 was found to play an important role in colorectal cancer cell proliferation and immune regulation, CMTR1, itself, is not currently targetable and its function in normal cells is poorly understood. Therefore, the drugs targeting CMTR1 may have potential toxicities to normal cells, which may pose a certain degree of challenge to new drug combinations. In summary, we will continue to improve our understanding of the mechanisms by which CMTR1 regulates immune escape to develop a new combination treatment for CRC.

## Supplementary information

aj-checklist

Supplementary Material

Uncropped western blotting image

Authors Contribution Statement

Figure S1

Figure S2

Figure S3

Figure S4

Figure S5

Figure S6

Figure S7

## Data Availability

The authors confirm that the data supporting the findings of this study are available within the article and/or its supplementary material, or can be made available upon reasonable request. RNA-seq data were deposited in the Gene Expression Omnibus database under accession GSE220040. The following link has been created to allow review of record GSE220040 while it remains in private status https://www.ncbi.nlm.nih.gov/geo/query/acc.cgi?acc=GSE220040.
