# Supplementary material for: CMTR1 promotes colorectal cancer cell growth and immune evasion by transcriptionally regulating STAT3
Source: Cell Death Dis. 2023 Apr 6;14(4):245. doi: 10.1038/s41419-023-05767-3 (PMC10079662; doi:10.1038/s41419-023-05767-3)
Supplement: Supplementary file 2 — Supplementary Material [file 41419_2023_5767_MOESM2_ESM.doc]

**Supplementary information**

**Supplementary figure legends**

**Figure S1: CMTR1 regulated tumor cell proliferation and immune response in HCT116 cells.**

(A-D) Expression of CDKN1A, CDK6 and CCND1 were determined by RT‒qPCR in HCT116 cells after CMTR1 knockdown.

(E) Expression of CDKN1A, CDK6，CCND1，pSTAT3 and STAT3 were determined by western blotting in HCT116 cells after CMTR1 knockdown.

(F) Expression of STAT3 was determined by RT‒qPCR in HCT116 cells after CMTR1 knockdown.

(G, H) CCK8 and colony formation assays were performed to detect cell proliferation in HCT116 cells.

(I-L) Relative mRNA levels of inflammatory mediators after knockdown of CMTR1 in HCT116 cells.

*P < 0.05, **P < 0.01, ***P < 0.001.

**Figure S2: CMTR1 regulated tumor cell proliferation and immune response in SW480 cells.**

(A-D) Expression of CDKN1A, CDK6 and CCND1 were determined by RT‒qPCR in SW480 cells after CMTR1 overexpression.

(E) Expression of CDKN1A, CDK6，CCND1，pSTAT3 and STAT3 were determined by western blotting in SW480 cells after CMTR1 overexpression.

(F) Expression of STAT3 was determined by RT‒qPCR in SW480 cells after CMTR1 overexpression.

(G, H) CCK8 and colony formation assays were performed to detect cell proliferation in SW480 cells.

(I-L) Relative mRNA levels of inflammatory mediators after overexpression of CMTR1 in SW480 cells.

*P < 0.05, **P < 0.01, ***P < 0.001.

**Figure S3:** **CMTR1 regulated cell cycle progression.**

1. Flow-cytometry analysis of RKO cells transfected with control or CMTR1 siRNAs and

stained with propidium iodide.

1. Flow-cytometry analysis of LoVo cells transfected with control or CMTR1-overexpressing plasmid and stained with propidium iodide.

**Figure S4:**

(A-C) CCK8 and colony formation assays were performed to detect cell proliferation in LoVo cells after CMTR1 knockdown.

(D-E) CCK8 and colony formation assays were performed to detect cell proliferation in MC38 cells after CMTR1 knockdown.

**Figure S5:**

(A, B) The CMTR1 protein levels in CRC tumor tissues (T) and matched adjacent normal tissues (N) were measured by western blotting (Replication 2 and Replication 3).

**Figure S6:**

(A, B)Expression of CDKN1A, CDK6，CCND1，pSTAT3 and STAT3 were determined by western blotting in RKO cells after CMTR1 knockdown (Replication 2 and Replication 3).

(C, D)Expression of CDKN1A, CDK6，CCND1，pSTAT3 and STAT3 were determined by western blotting in HCT116 cells after CMTR1 knockdown (Replication 2 and Replication 3).

**Figure S7:**

(A, B)Expression of CDKN1A, CDK6，CCND1，pSTAT3 and STAT3 were determined by western blotting in LoVo cells after CMTR1 overexpression (Replication 2 and Replication 3).

(C, D)Expression of CDKN1A, CDK6，CCND1，pSTAT3 and STAT3 were determined by western blotting in SW480 cells after CMTR1 overexpression (Replication 2 and Replication 3).
