# Supplementary material for: CMTR1 promotes colorectal cancer cell growth and immune evasion by transcriptionally regulating STAT3
Source: Cell Death Dis. 2023 Apr 6;14(4):245. doi: 10.1038/s41419-023-05767-3 (PMC10079662; doi:10.1038/s41419-023-05767-3)

Figure 1E-H

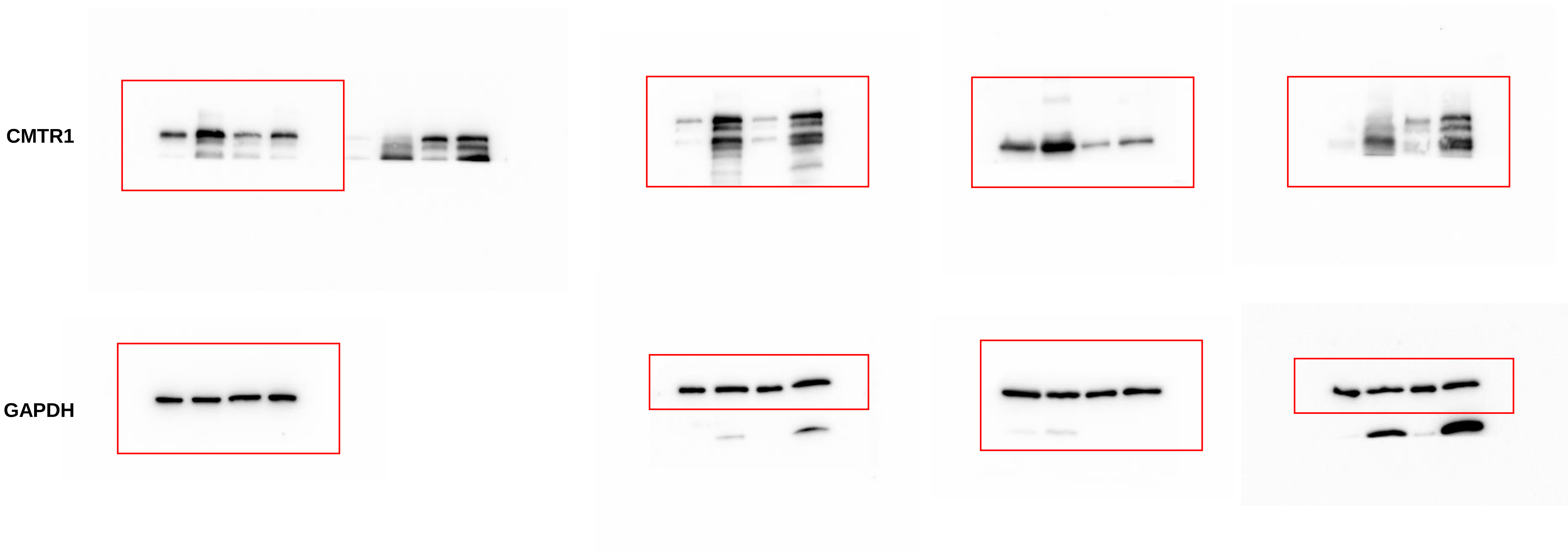

Figure 2P

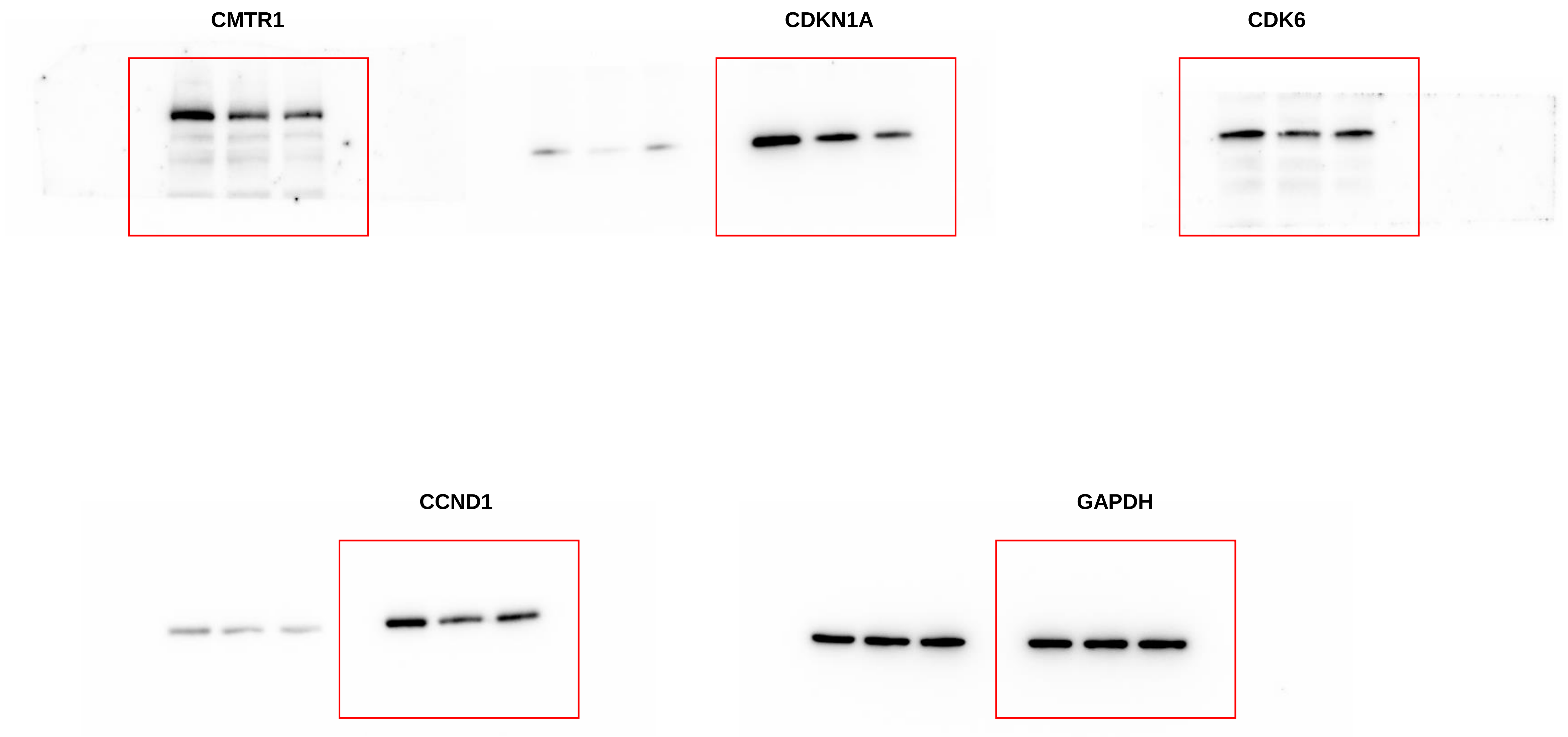

Figure 2Q

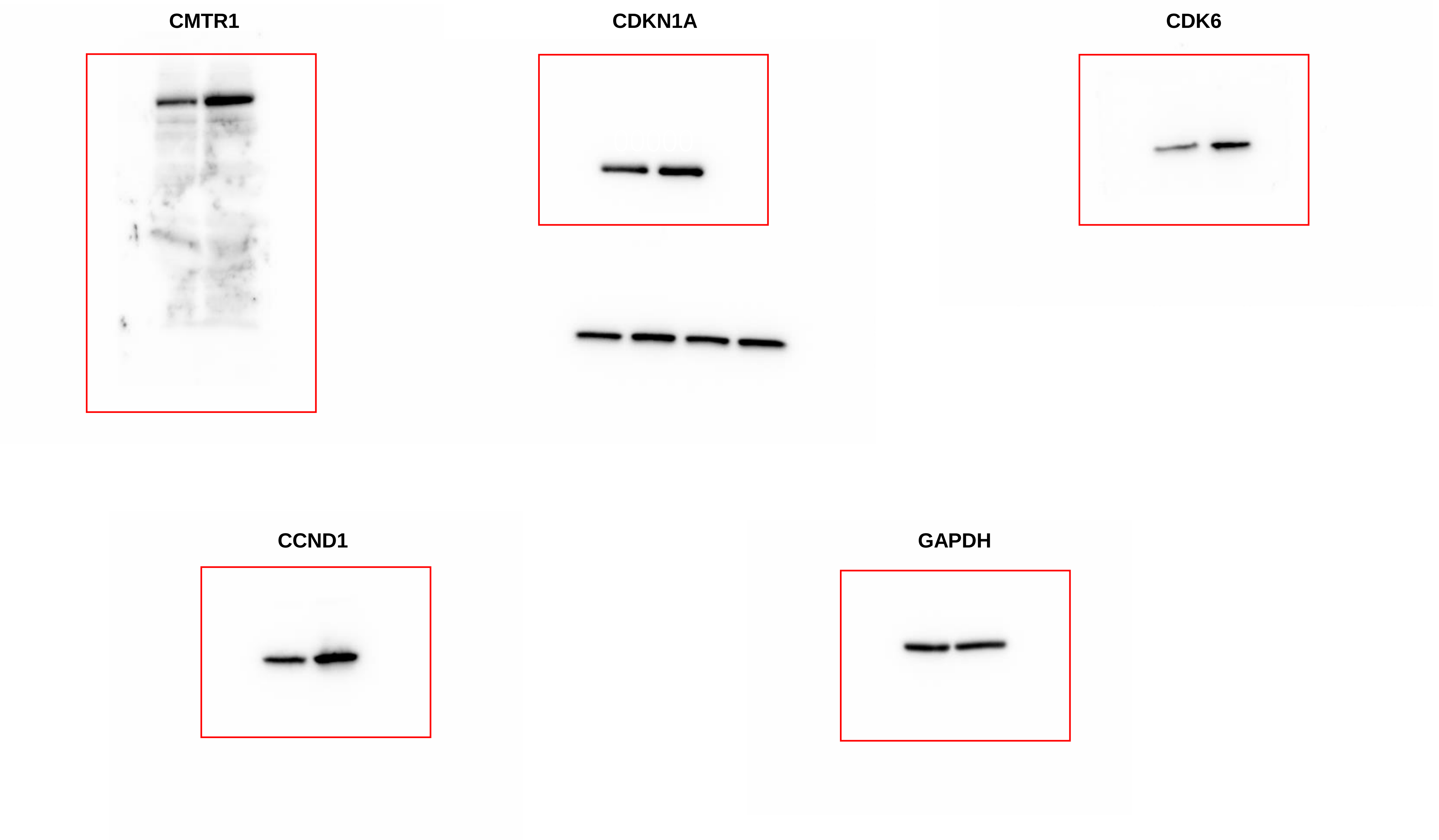

Figure 4D

pSTAT3

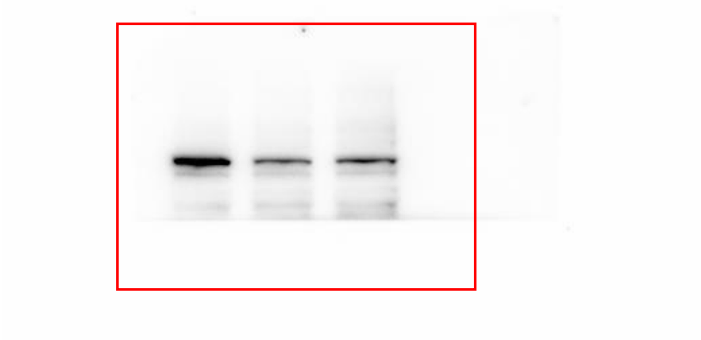

STAT3

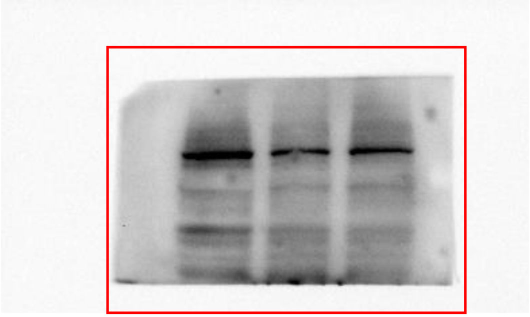

GAPDH

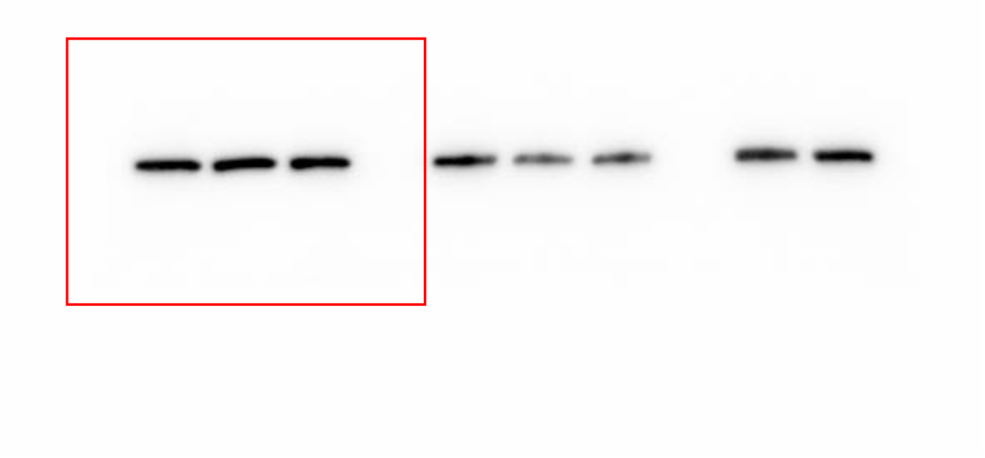

Figure 4E

pSTAT3

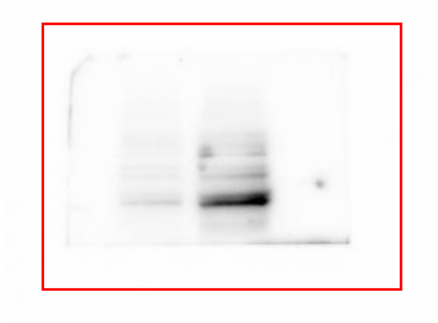

STAT3

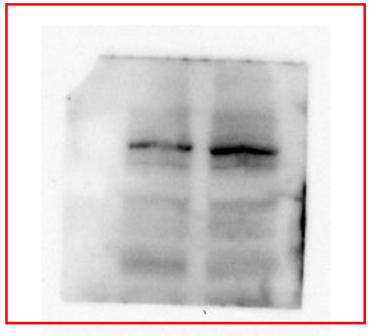

GAPDH

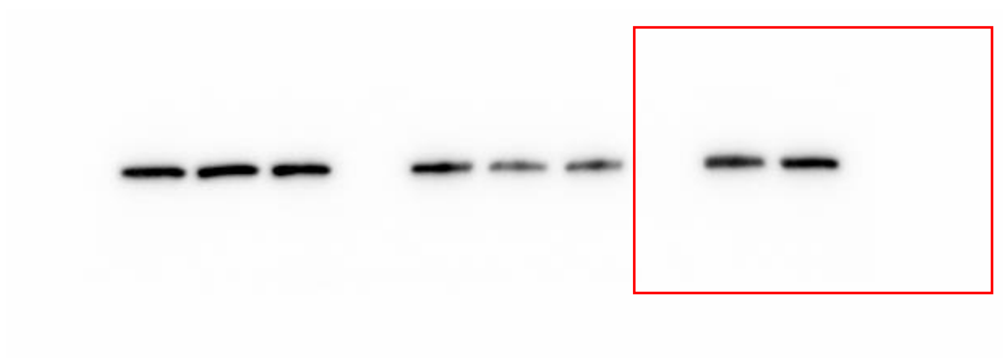

Figure 5C

CMTR1 STAT3 GAPDH

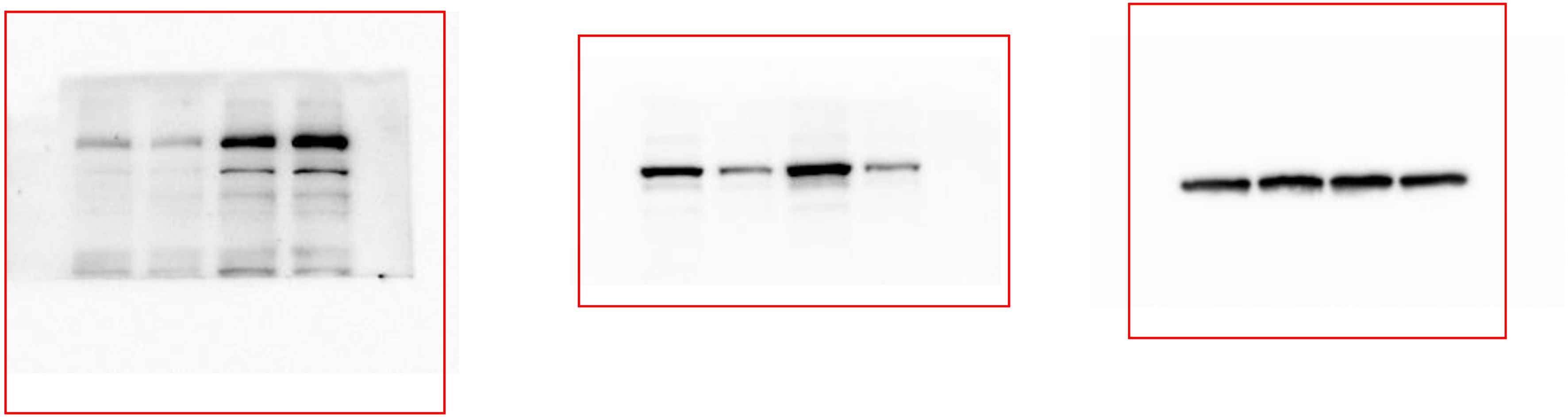

Figure 6C

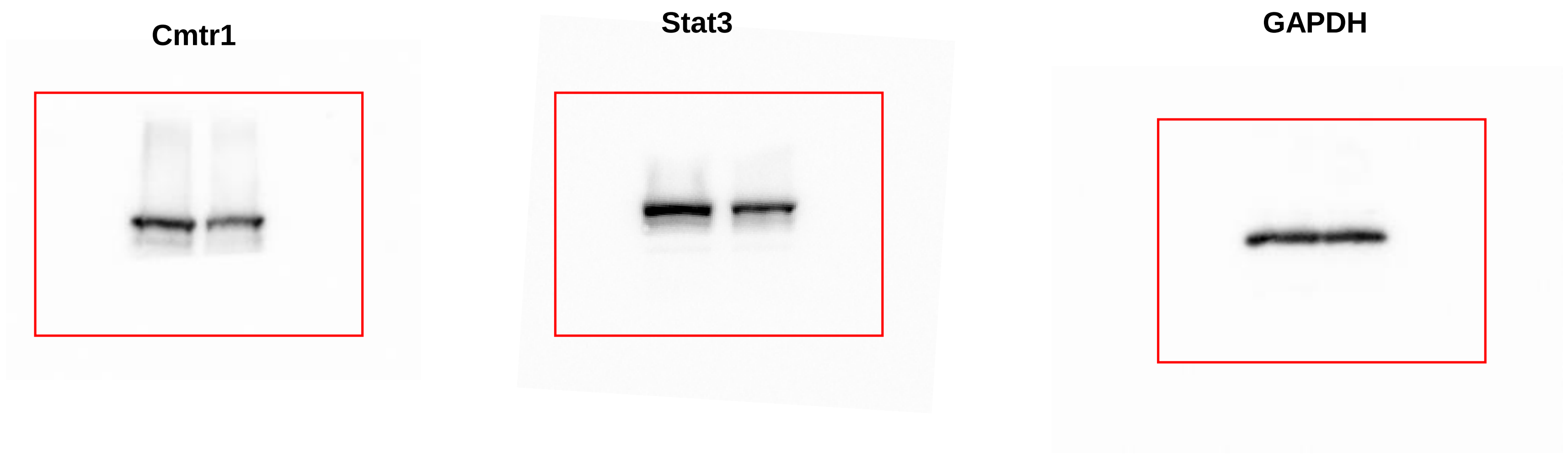

Figure S1

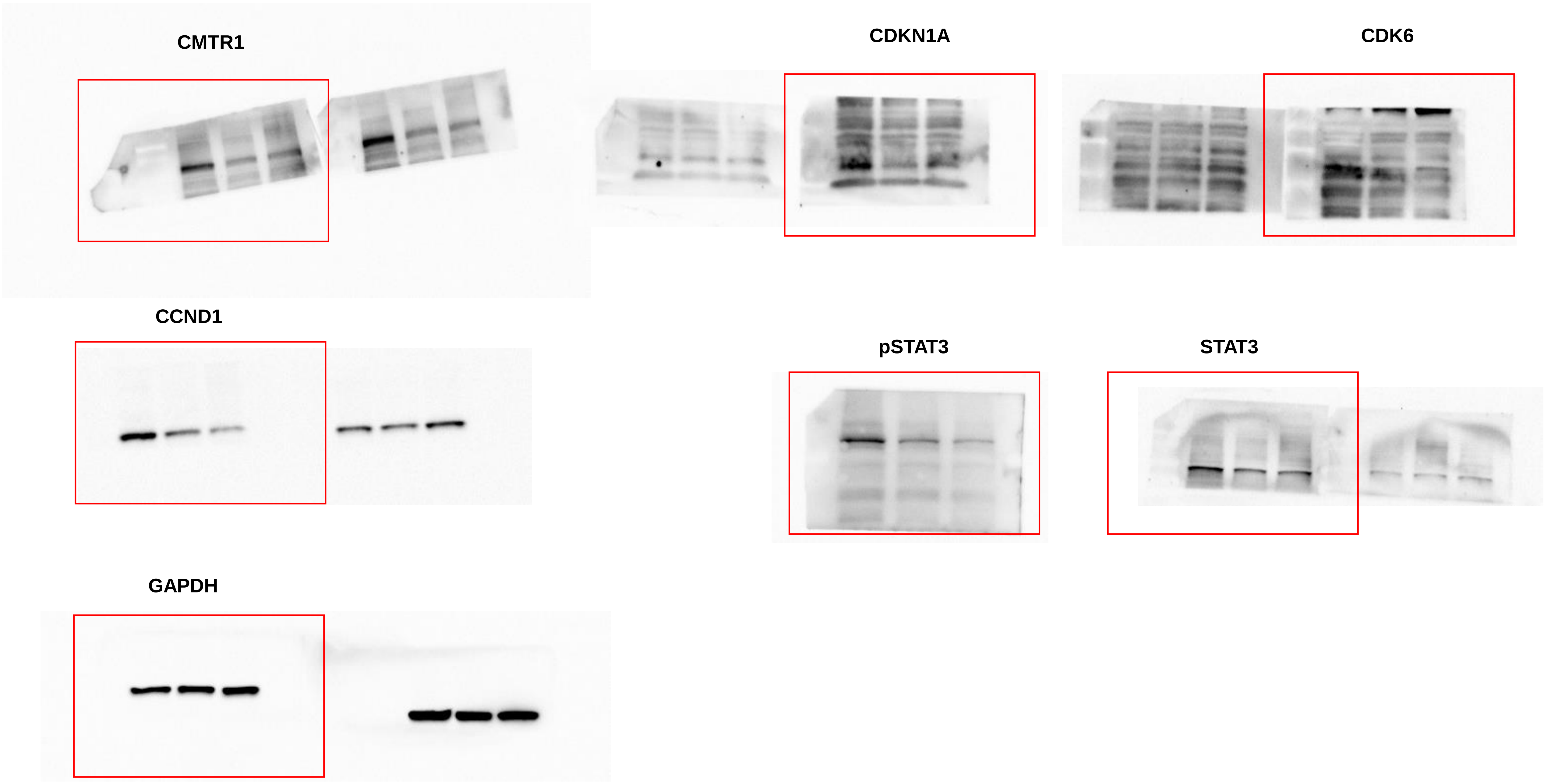

Figure S2

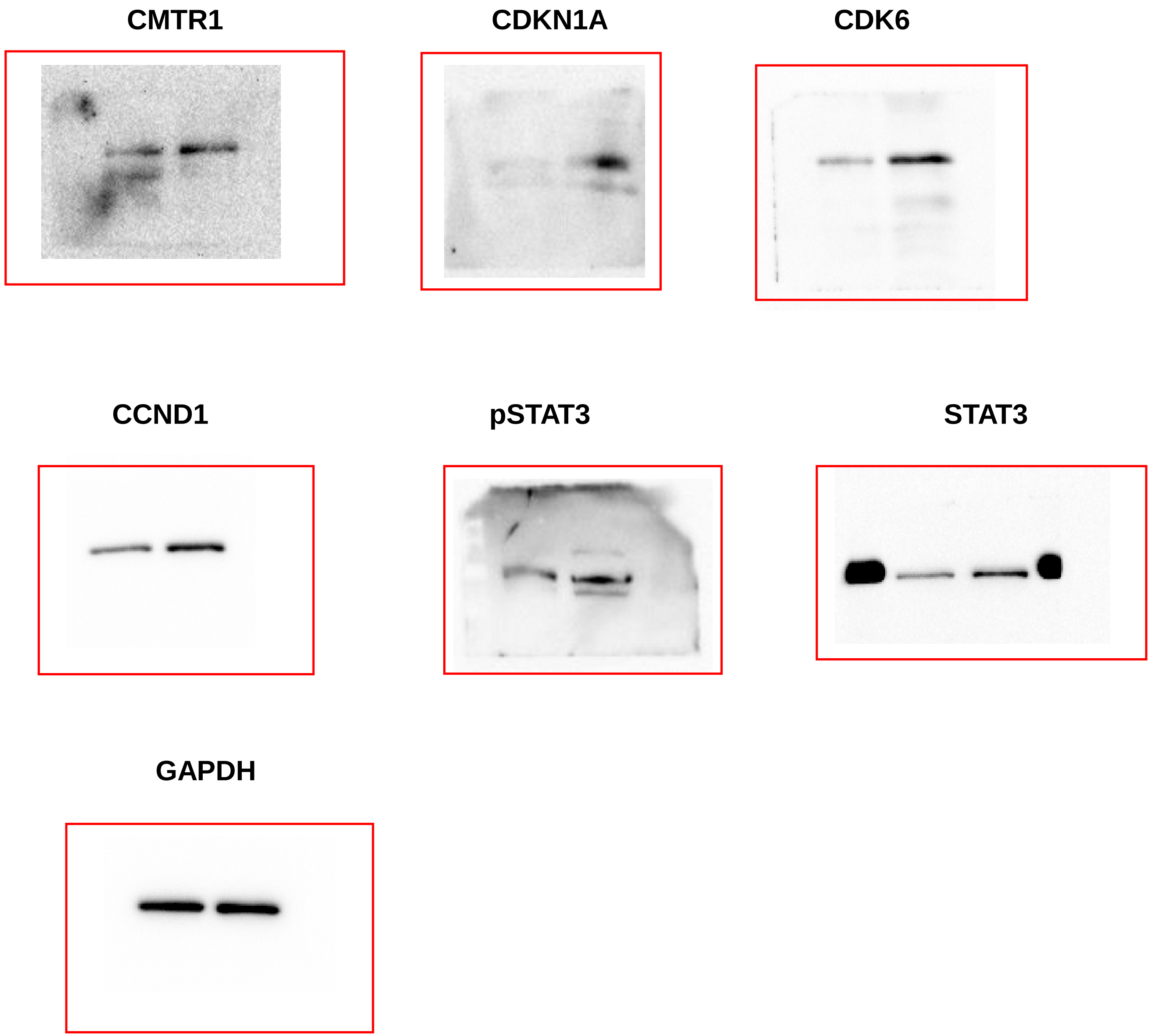

Figure S5A

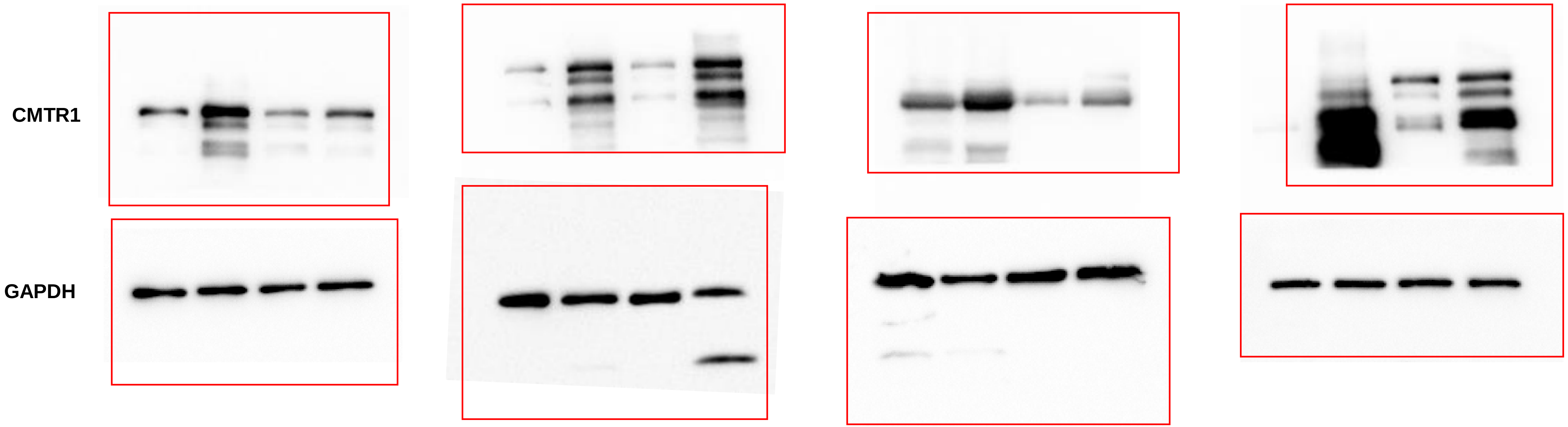

Figure S5B

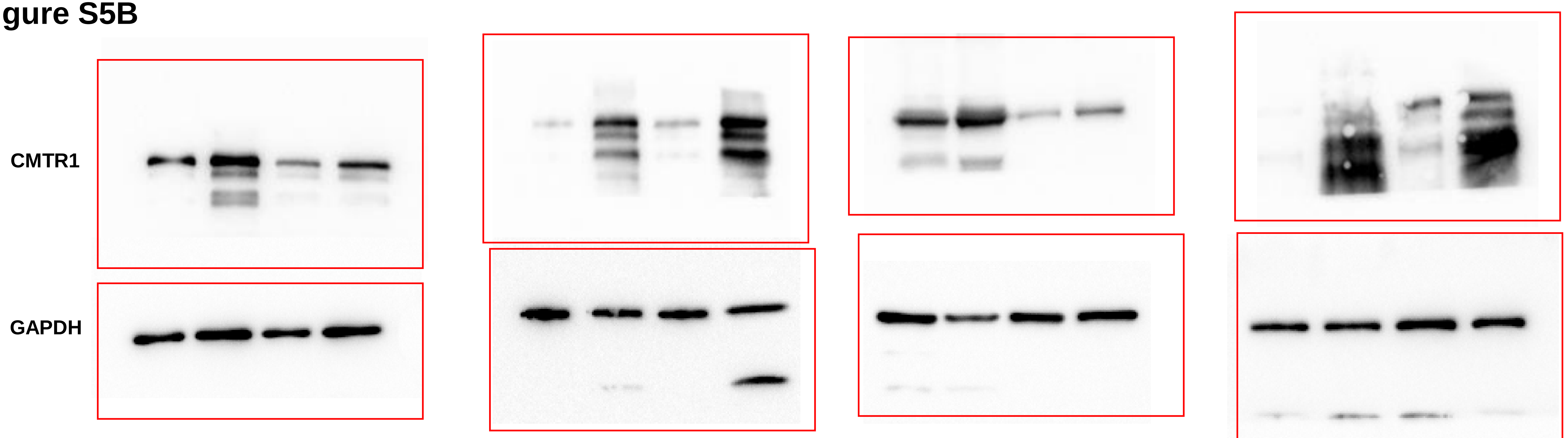

Figure S6A

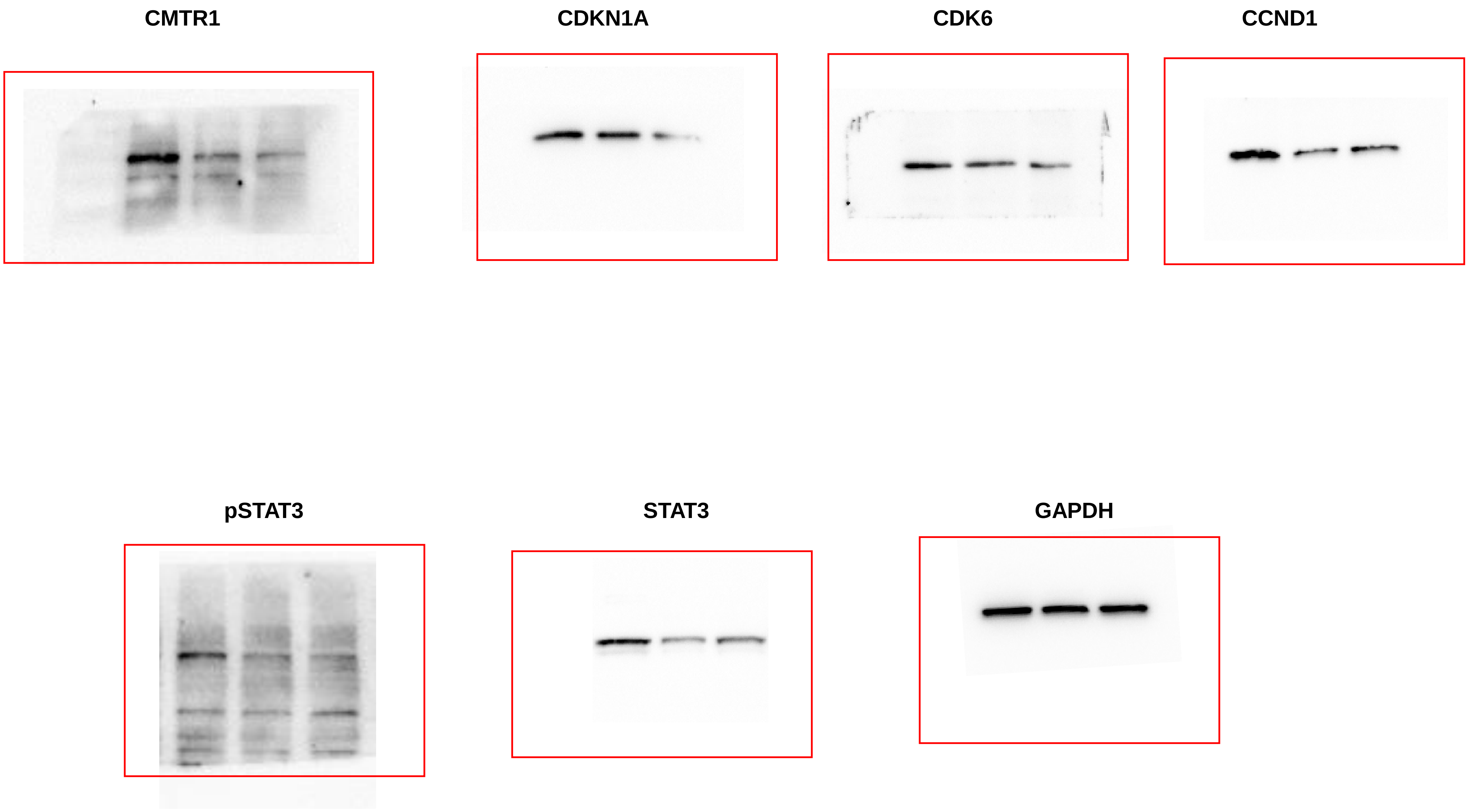

Figure S6B

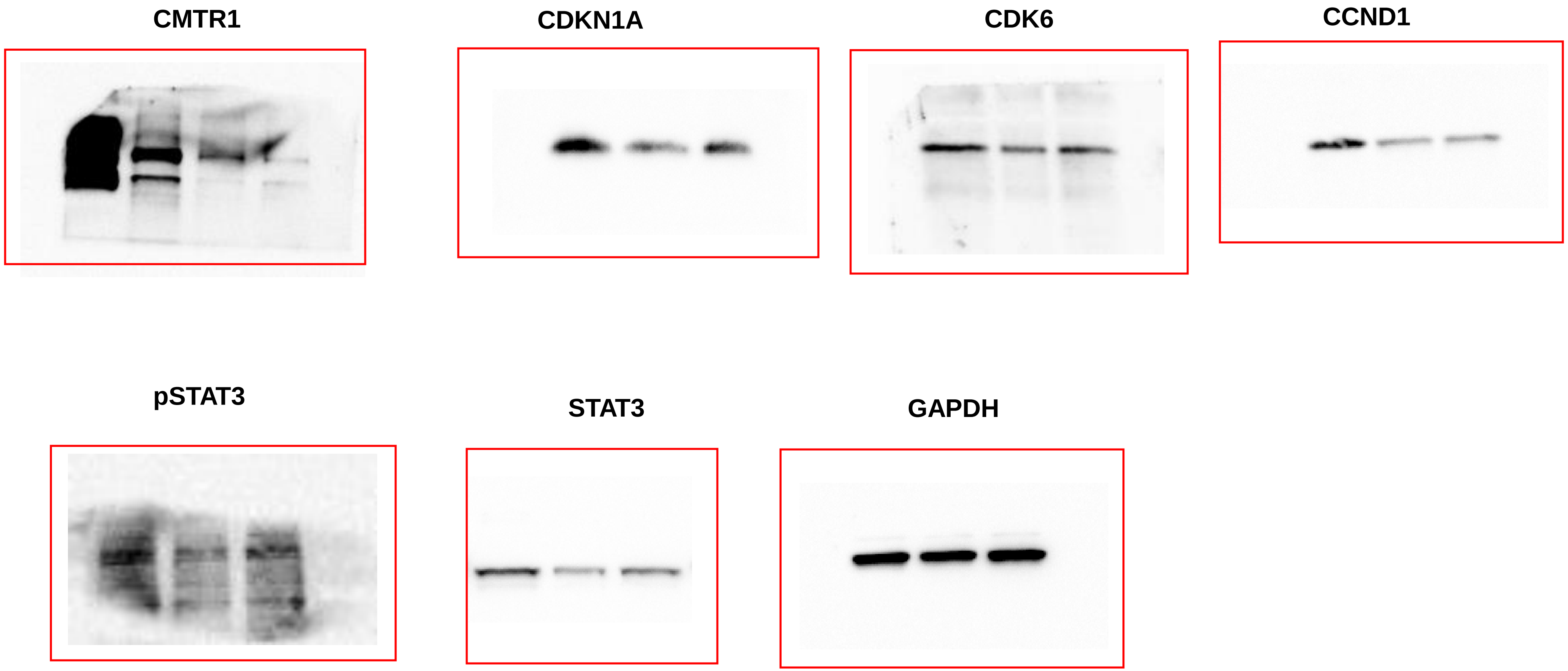

Figure S6C

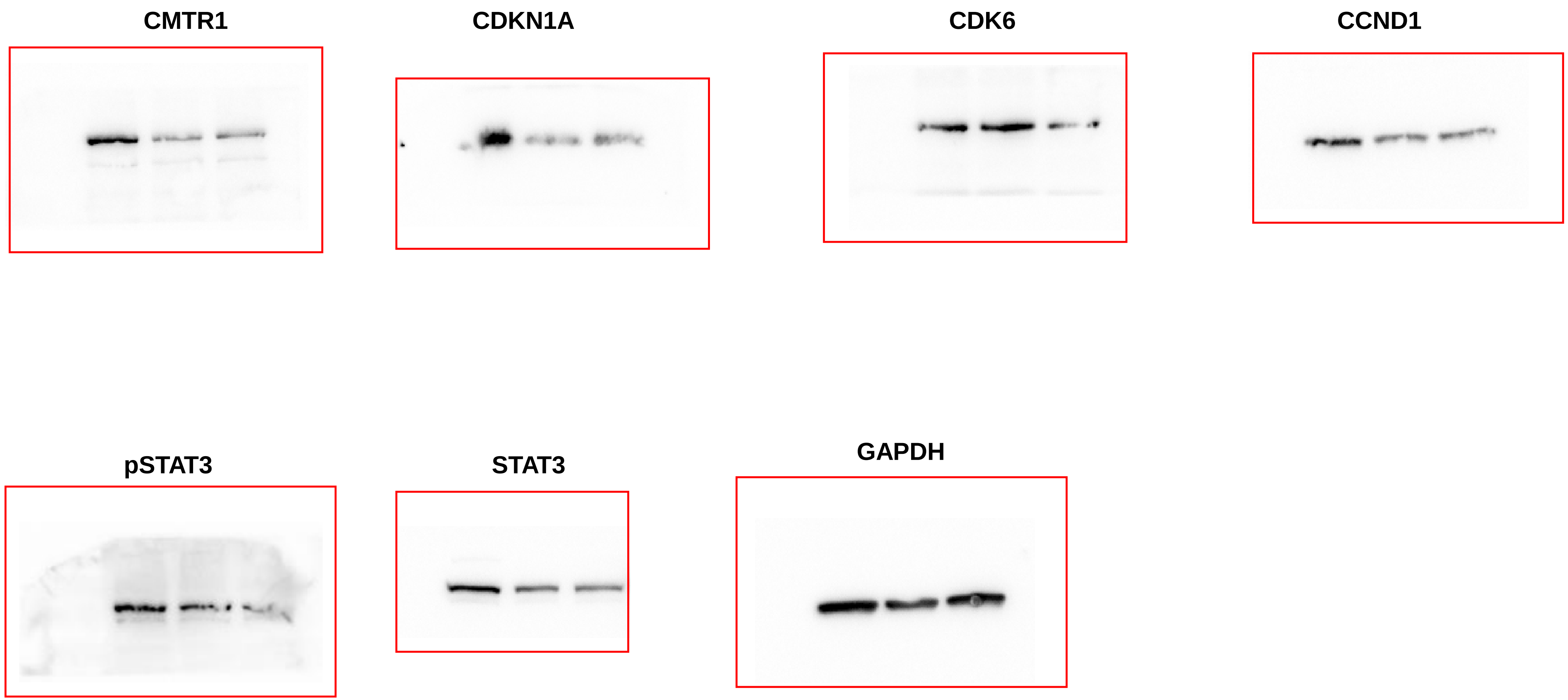

Figure S6D

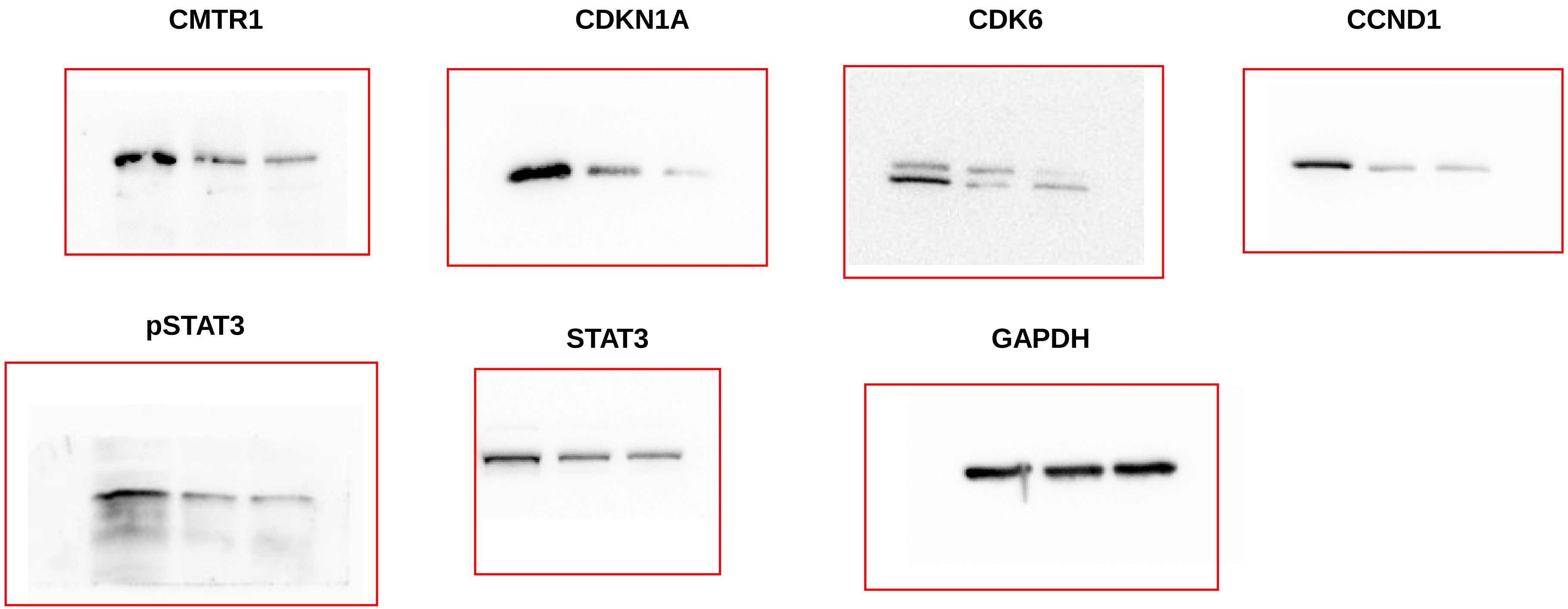

Figure S7A

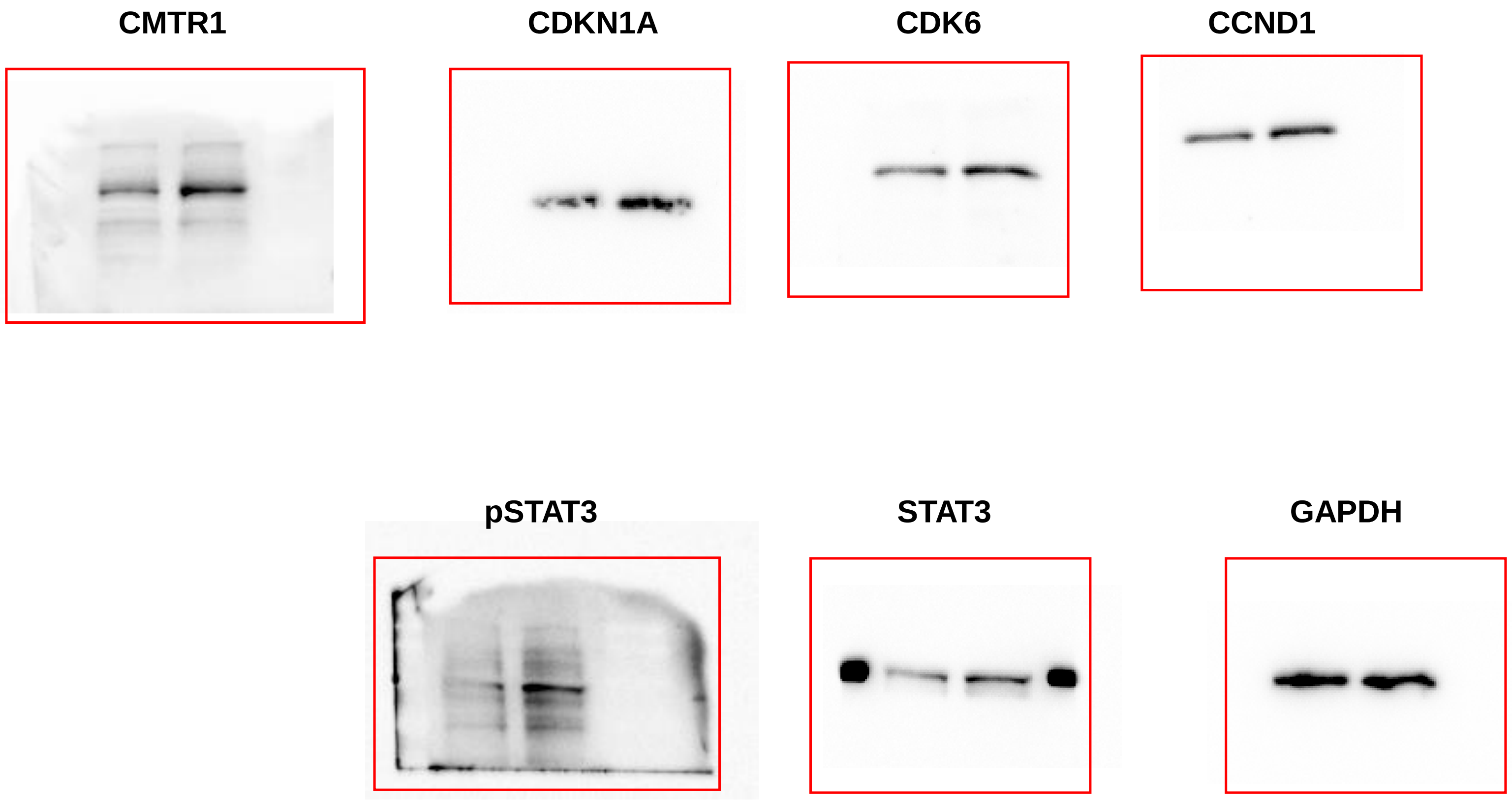

Figure S7B

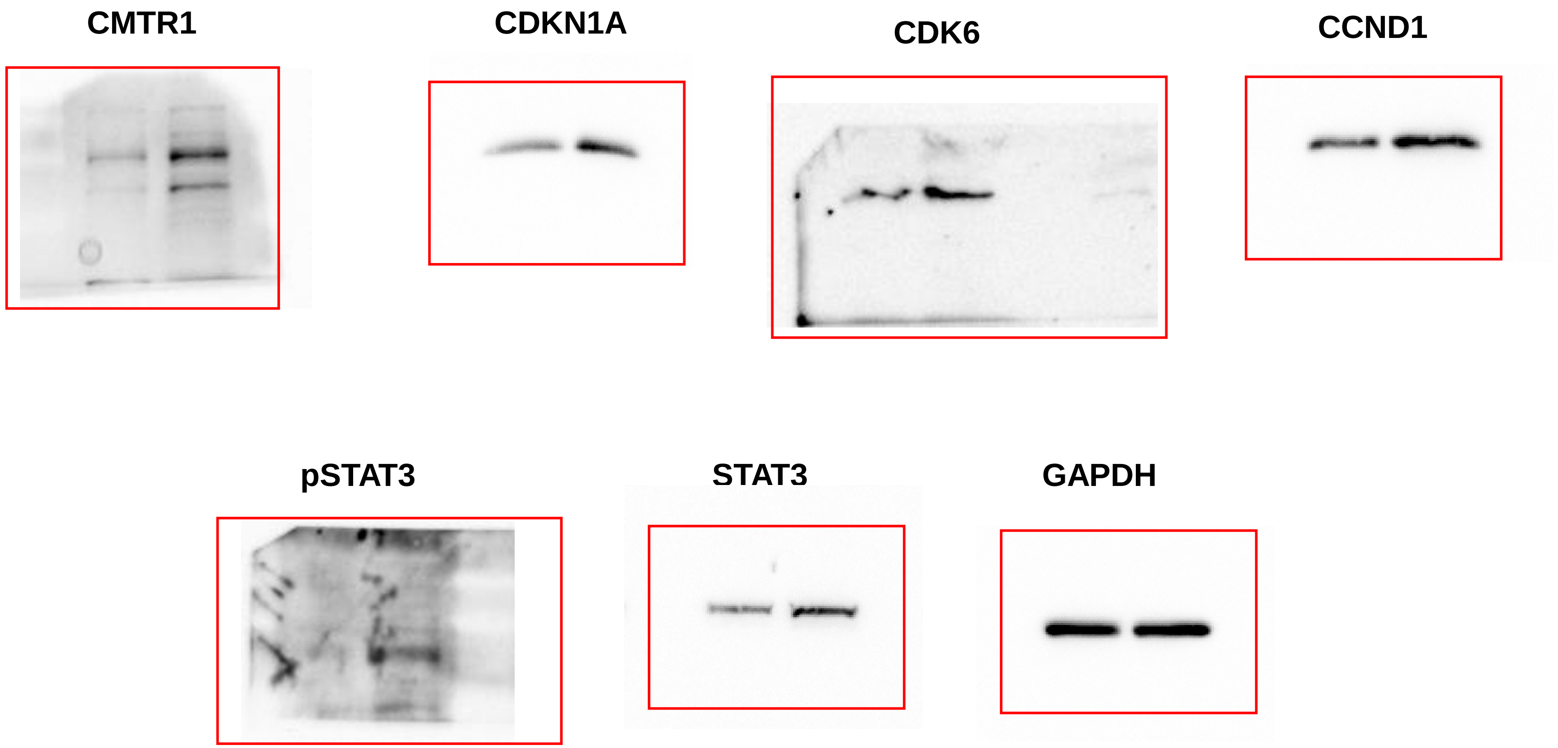

Figure S7C

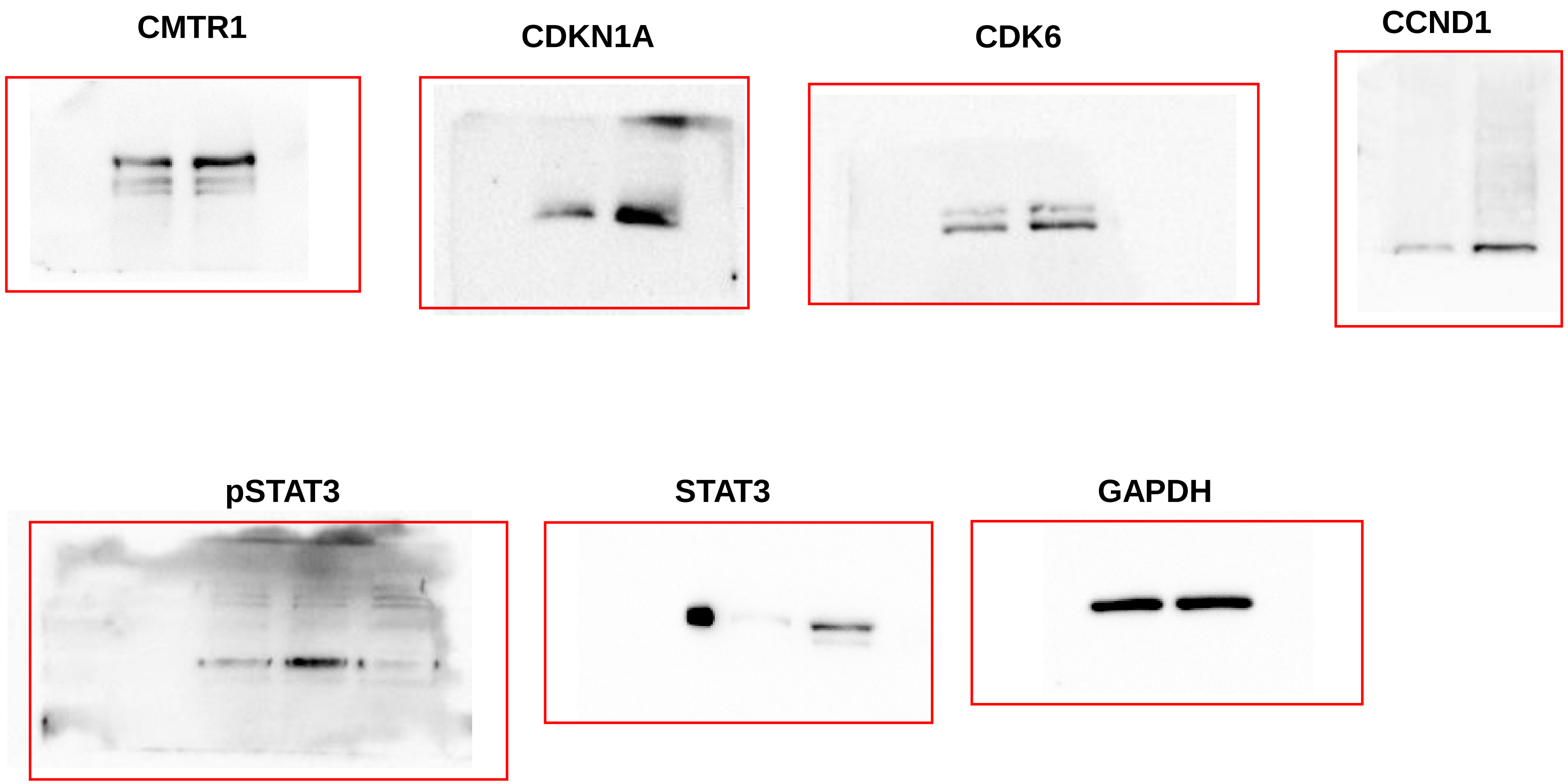

Figure S7D

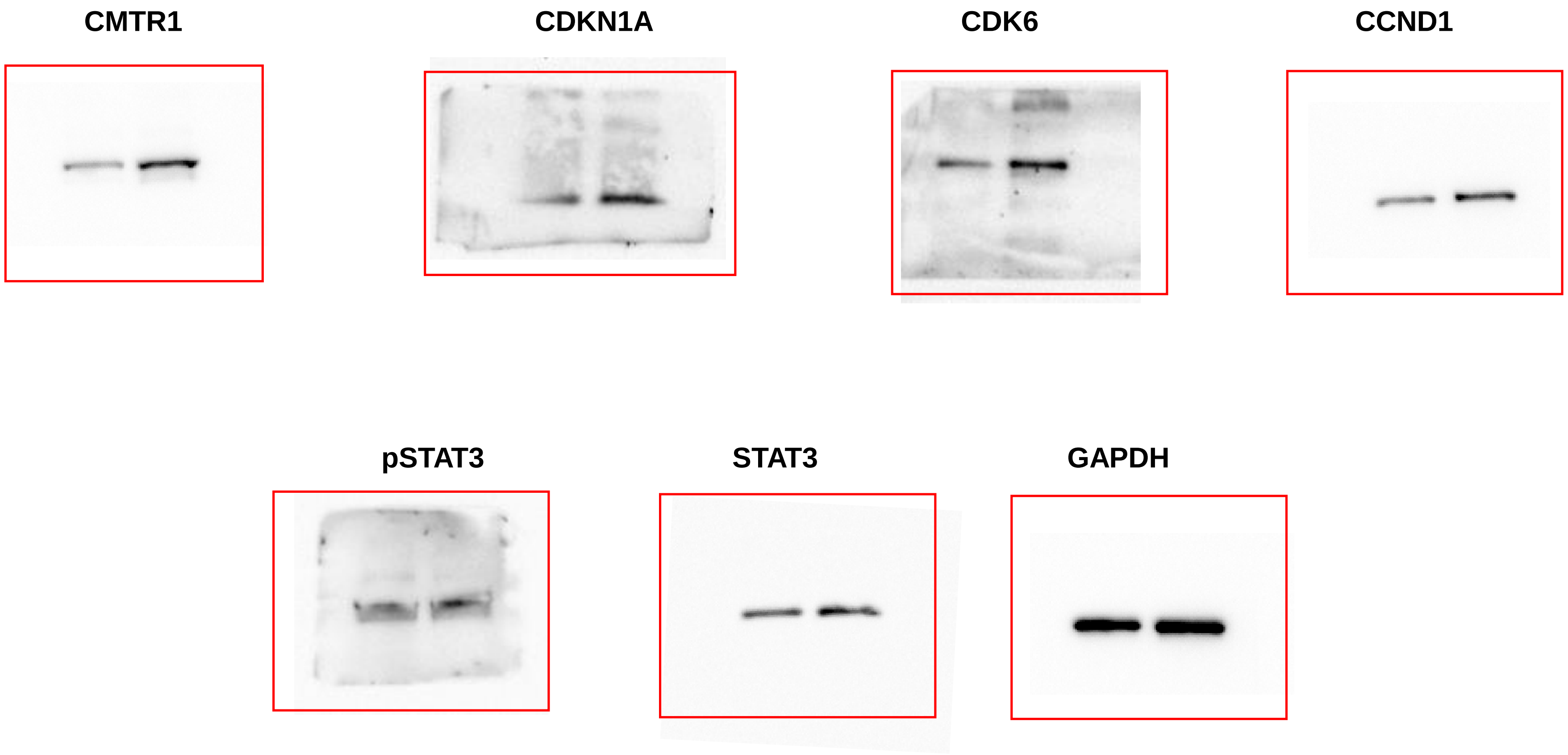

Supplement: Supplementary file 3 — Uncropped western blotting image [file 41419_2023_5767_MOESM3_ESM.pdf]
