# Supplementary material for: CMTR1 promotes colorectal cancer cell growth and immune evasion by transcriptionally regulating STAT3
Source: Cell Death Dis. 2023 Apr 6;14(4):245. doi: 10.1038/s41419-023-05767-3 (PMC10079662; doi:10.1038/s41419-023-05767-3)
Supplement: Supplementary file 4 — Authors Contribution Statement [file 41419_2023_5767_MOESM4_ESM.docx]

**Table S1** Sequences of oligonucleotides and primers

| Assay | Oligo name | Oligo sequence (5’-3’) |  |
| --- | --- | --- | --- |
| Primers used for RT-qPCR to check gene expression | CMTR1-F | AACGTGGACTGGCGAGATG |  |
|  | CMTR1-R | CCAATCGCTCATTTCCTGAGT |  |
|  | CDKN1A-F | TGTCCGTCAGAACCCATGC |  |
|  | CDKN1A-R | AAAGTCGAAGTTCCATCGCTC |  |
|  | CDK6-F | TCTTCATTCACACCGAGTAGTGC |  |
|  | CDK6-R | TGAGGTTAGAGCCATCTGGAAA |  |
|  | CCND1-F | GCTGCGAAGTGGAAACCATC |  |
|  | CCND1-R | CCTCCTTCTGCACACATTTGAA |  |
|  | CXCL9-F | CCAGTAGTGAGAAAGGGTCGC |  |
|  | CXCL9-R  CXCL10-F  CXCL10-R  TNFα-F  TNFα-R  IFNα-F  IFNα-R  STAT3-F  STAT3-R  GAPDH-F  GAPDH-R  Mus-Cxcl9-F  Mus-Cxcl9-R  Mus-Cxcl10-F  Mus-Cxcl10-R  Mus-Tnfα-F  Mus-Tnfα-R  Mus-Ifnα-F  Mus-Ifnα-R  Mus-Gapdh-F  Mus-Gapdh-R | AGGGCTTGGGGCAAATTGTT  GTGGCATTCAAGGAGTACCTC  TGATGGCCTTCGATTCTGGATT  CCTCTCTCTAATCAGCCCTCTG  GAGGACCTGGGAGTAGATGAG  GCCTCGCCCTTTGCTTTACT  CTGTGGGTCTCAGGGAGATCA  CAGCAGCTTGACACACGGTA  AAACACCAAAGTGGCATGTGA  GAAGGTGAAGGTCGGAGT  GAGGATGGTGATGGGATTTC  TCCTTTTGGGCATCATCTTCC  TTTGTAGTGGATCGTGCCTCG  CCAAGTGCTGCCGTCATTTTC  GGCTCGCAGGGATGATTTCAA  CTGAACTTCGGGGTGATCGG  GGCTTGTCACTCGAATTTTGAGA  GATTCCCGCAGGAGAAGGTG  TCCATGCAGCAGATGAGTCC  AGGTCGGTGTGAACGGATTTG  TGTAGACCATGTAGTTGAGGTCA |  |
| ChIP-qPCR primers targeting STAT3  promoter | STAT3 promoter P1-F | AGGGGCGTATCAGTCTCCTC |  |
|  | STAT3 promoter P1-R | ATTCATGAAAGGCCAGCTCGT |  |
|  | STAT3 promoter P2-F | CGAACGAGCTGGCCTTTCAT |  |
|  | STAT3 promoter P2-R | TCCCCCTCCCAGTCTGC |  |
| siRNA or shRNA | si-CMTR1#1 | GAACGAGTTTGTCTCTTCA |  |
|  | si-CMTR1#2  si-STAT3#1  si-STAT3#2  sh-CMTR1  Mus-sh-Cmtr1 | CGAGGAGTCTTCTTTCTAA  GGCGTCCAGTTCACTACTA  AGACCCGTCAACAAATTAA  GAACGAGTTTGTCTCTTCA  GCGAGGGATTGGGTAAATACA |  |
