# Supplementary figures and images for: CMTR1 promotes colorectal cancer cell growth and immune evasion by transcriptionally regulating STAT3
Source: Cell Death Dis. 2023 Apr 6;14(4):245. doi: 10.1038/s41419-023-05767-3 (PMC10079662; doi:10.1038/s41419-023-05767-3)

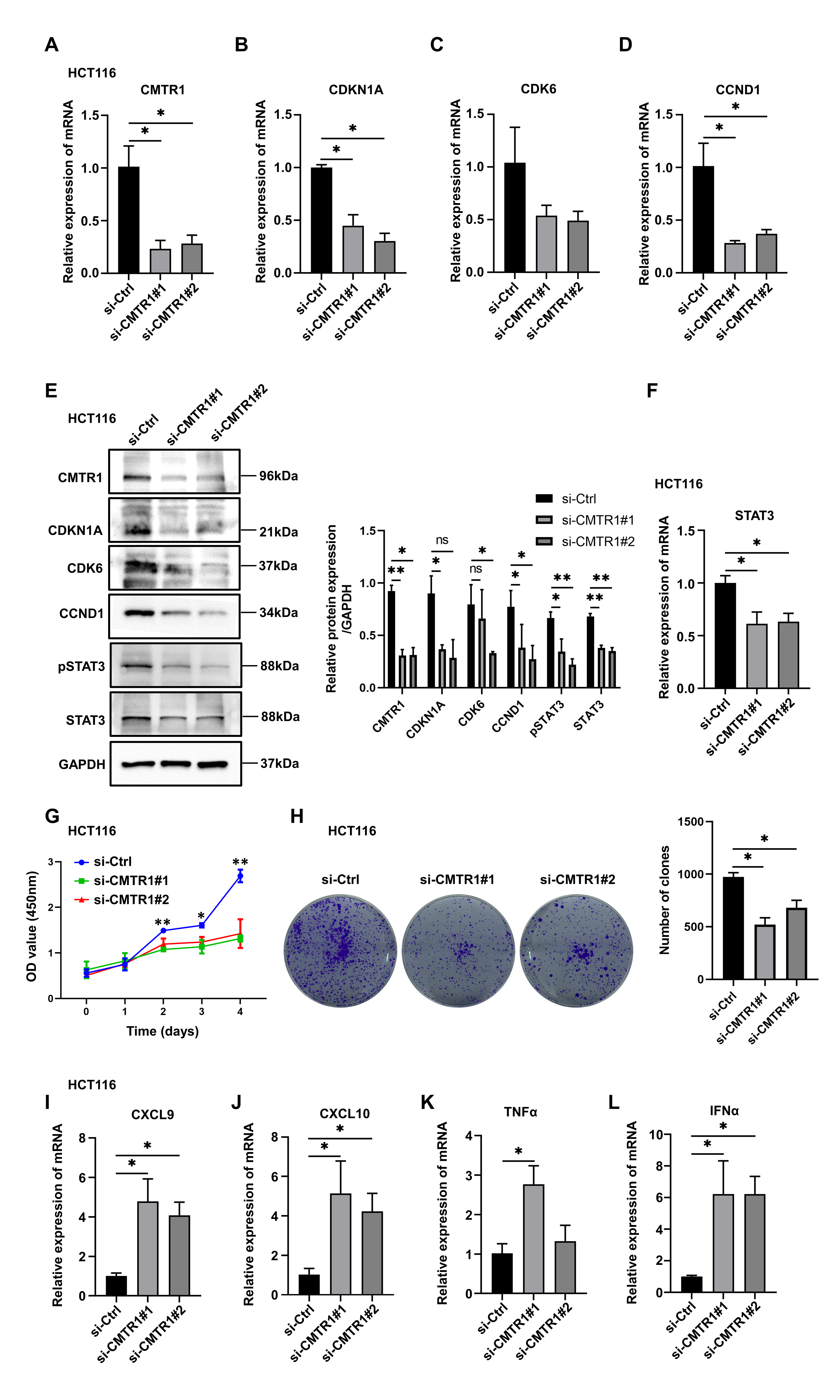

Supplement: Supplementary file 5 — Figure S1 [file 41419_2023_5767_MOESM5_ESM.tif]

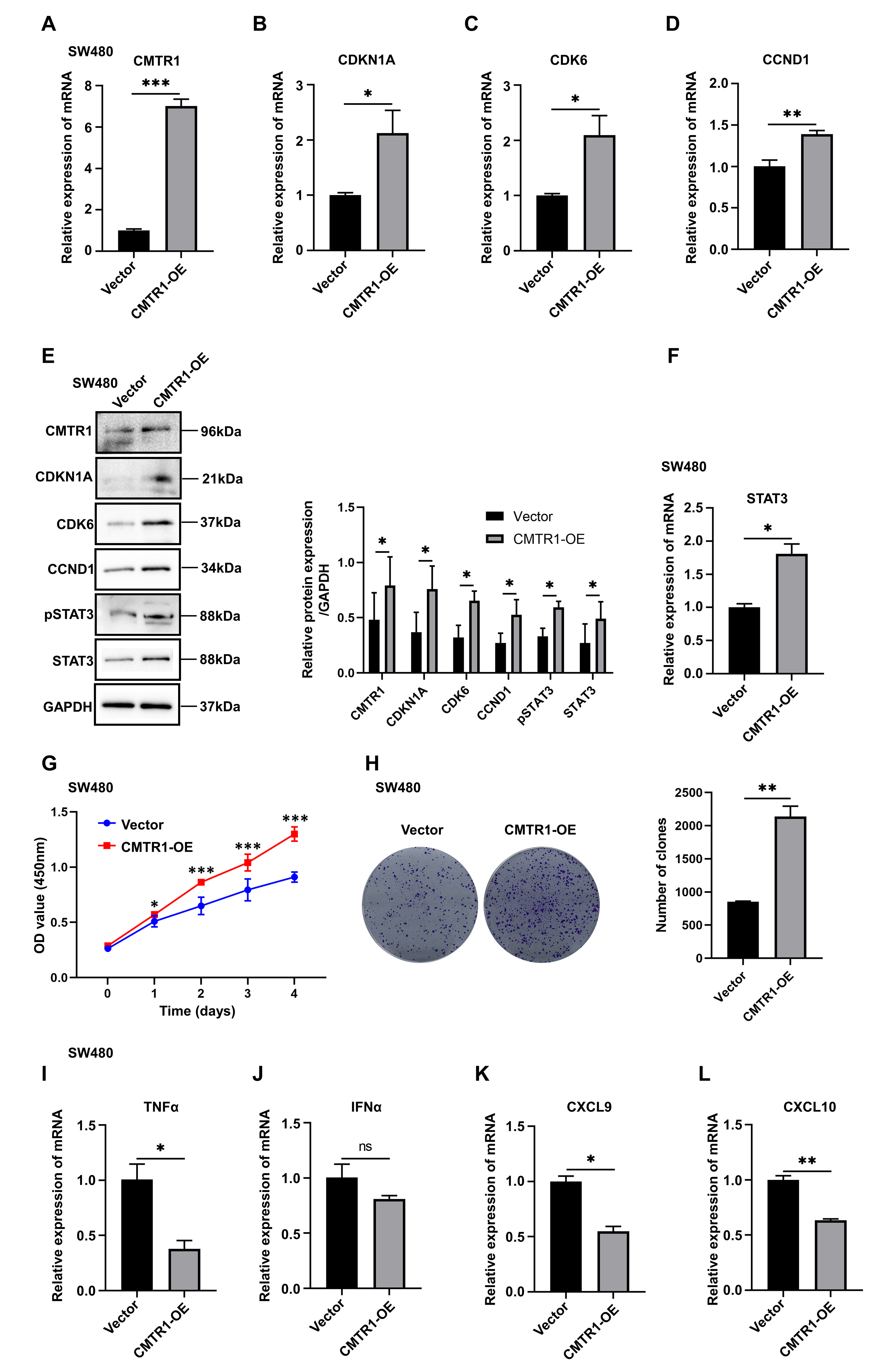

Supplement: Supplementary file 6 — Figure S2 [file 41419_2023_5767_MOESM6_ESM.tif]

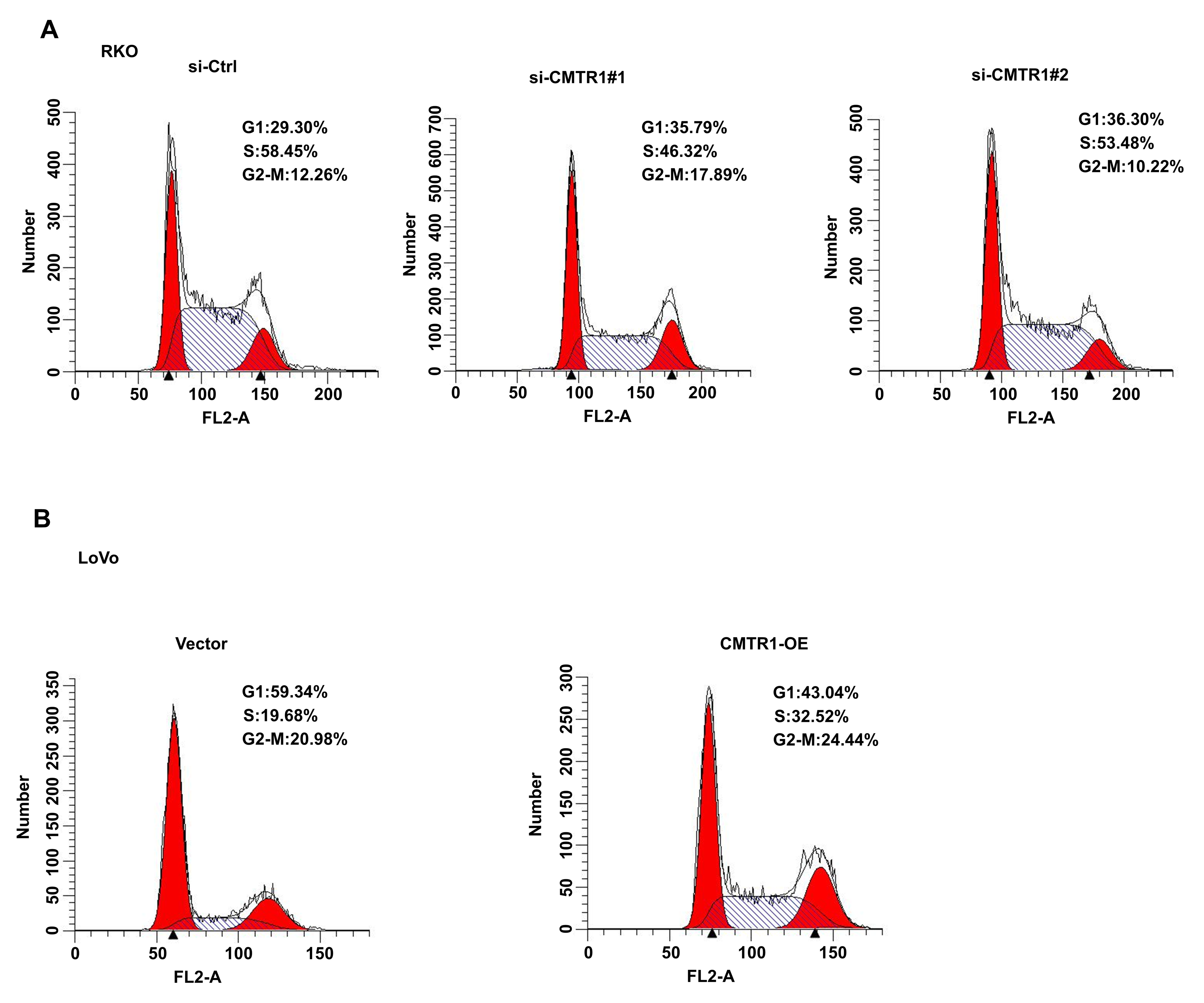

Supplement: Supplementary file 7 — Figure S3 [file 41419_2023_5767_MOESM7_ESM.tif]

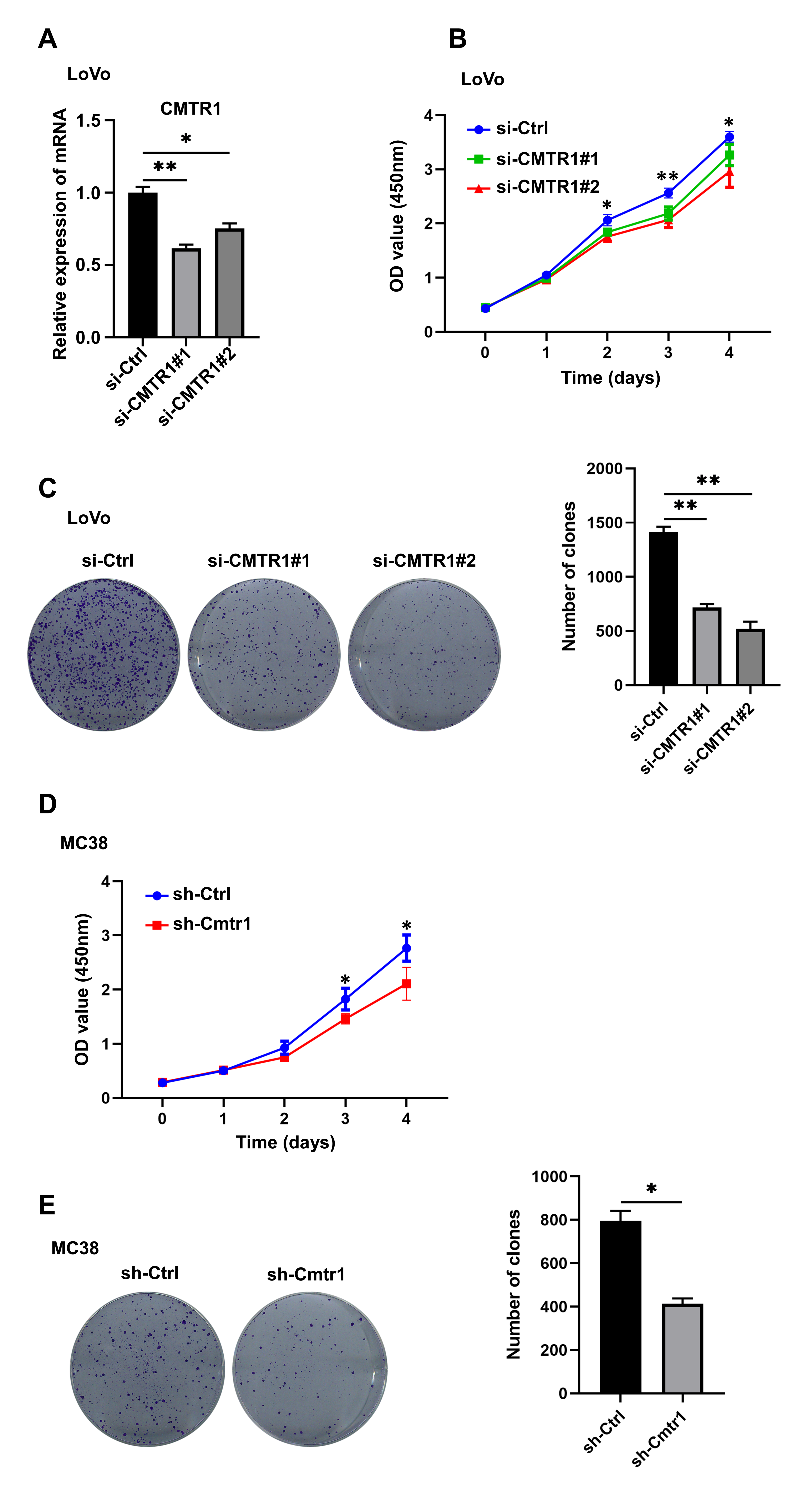

Supplement: Supplementary file 8 — Figure S4 [file 41419_2023_5767_MOESM8_ESM.tif]

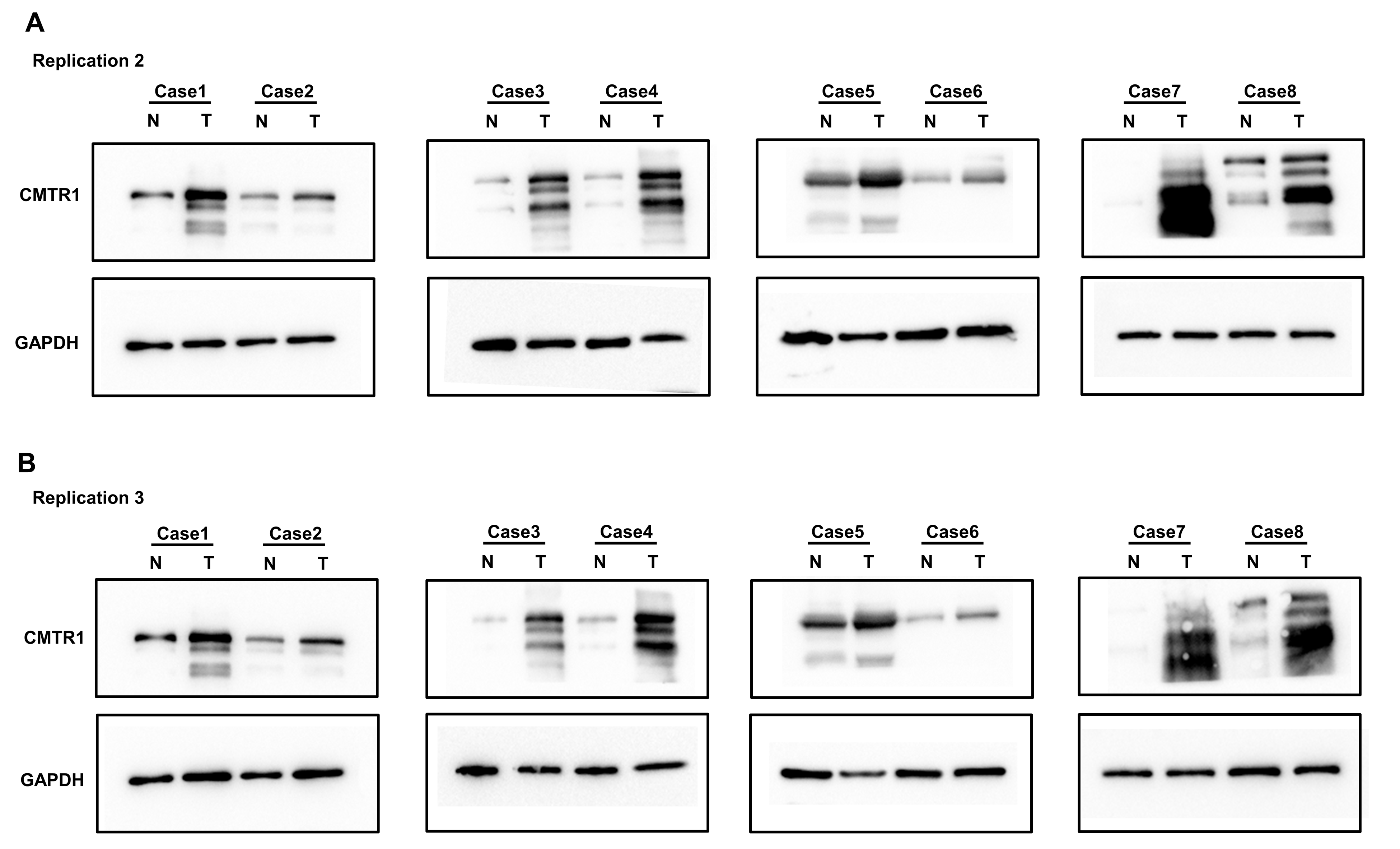

Supplement: Supplementary file 9 — Figure S5 [file 41419_2023_5767_MOESM9_ESM.tif]

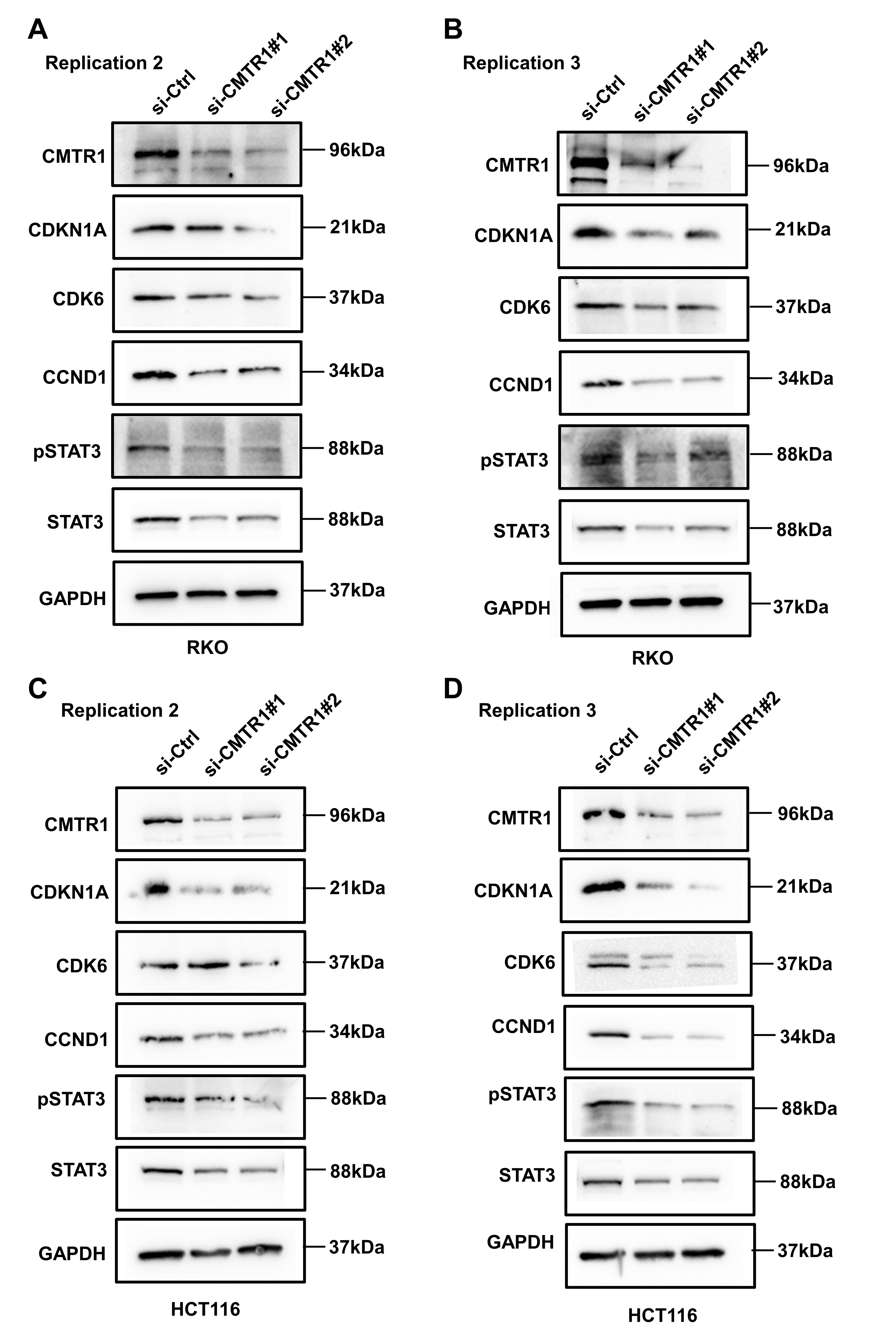

Supplement: Supplementary file 10 — Figure S6 [file 41419_2023_5767_MOESM10_ESM.tif]

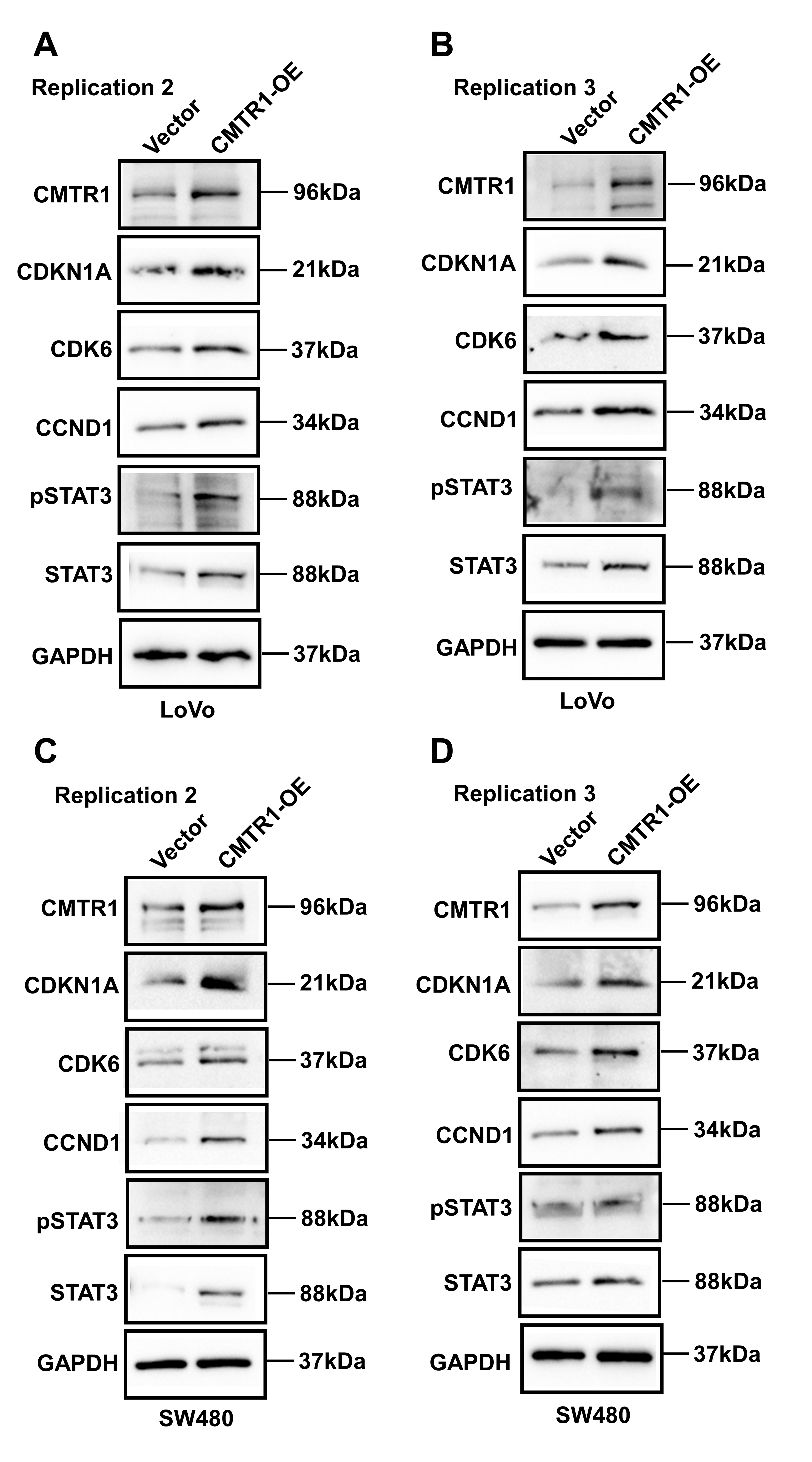

Supplement: Supplementary file 11 — Figure S7 [file 41419_2023_5767_MOESM11_ESM.tif]
